# A mathematical model of GLUT1 modulation in rods and RPE and its differential impact in cell metabolism

**DOI:** 10.1038/s41598-022-13950-3

**Published:** 2022-06-23

**Authors:** Andrea Aparicio, Erika T. Camacho, Nancy J. Philp, Stephen A. Wirkus

**Affiliations:** 1 School of Mathematical and Natural Sciences, Arizona State University, Glendale, AZ USA; 2 Department of Pathology, Anatomy and Cell Biology, Thomas Jefferson University, Philadelphia, PA USA

**Keywords:** Cell biology, Systems biology, Mathematics and computing

## Abstract

We present a mathematical model of key glucose metabolic pathways in two cells of the human retina: the rods and the retinal pigmented epithelium (RPE). Computational simulations of glucose transporter 1 (GLUT1) inhibition in the model accurately reproduce experimental data from conditional knockout mice and reveal that modification of GLUT1 expression levels of both cells differentially impacts their metabolism. We hypothesize that, under glucose scarcity, the RPE’s energy producing pathways are altered in order to preserve its functionality, impacting the photoreceptors’ outer segment renewal. On the other hand, when glucose is limited in the rods, aerobic glycolysis is preserved, which maintains the lactate contribution to the RPE.

## Introduction

Systems biology is transforming current approaches to biological research and therapeutic treatments. Mathematical models that accurately describe biological processes have become a standard tool to investigate the interconnectedness of complex regulatory processes and how disruptions of these processes may contribute to the development of disease^1–3^. Successful applications range from cancer and human immunodeficiency virus (HIV) research, energy metabolism analysis, and the design of drugs and assistive devices for their delivery, to the study of specific disease progression in the retina^4–13^. A mathematical model that accurately reproduces experimental data allows one to verify new hypotheses, analyze perturbations, and explore potential hypotheses and treatment options via computational simulations, yielding results quickly and at an extremely low cost. Numerical simulations provide a computationally informed perspective of the functioning of the system, key to the design of new experiments, shifting the paradigm of biological sciences.

The retina is the light-sensitive tissue of vertebrates’ eyes and serves as the sensor with which vision is enabled. It consists of various layers of interconnected cells. Here we provide an analysis of the interconnected mechanisms and metabolic processes of two cells located in the outer human retina: the retinal pigmented epithelium (RPE) and the rod photoreceptors. We focus on the role that glucose transporter 1 (GLUT1) plays in these functions.

The RPE is a structurally and functionally polarized epithelia^14^ that serves as a selective barrier in the retina, regulating the vectorial transport of nutrients into the retina, phagocytosis of shed outersegments, and visual pigment regeneration^15^. Glucose is the primary metabolic substrate of the neural retina and the RPE facilitates the transepithelial transport of glucose from the choriocapillaris to the outer retina via GLUT1^16^. The RPE’s apical surface faces the interphotoreceptor matrix, through which metabolites are exchanged with photoreceptor and Muller glial cells in the outer neural retina^17^.

Photoreceptors convert light into electric impulses that are transmitted to the brain for image interpretation. They are sensory epithelium with outer segment connected to the inner segment via a connecting cilium. The outer segments are comprised of stacks of membrane discs that have a high concentration of the light sensitive protein opsin bound to 11-cis retinal. There are two main kinds of photoreceptors: rods and cones. The rods are extremely sensitive to light and are responsible for night vision. Cones are relatively insensitive to light and are responsible for high acuity central vision and color vision^18^. Photoreceptors are among the most energy-demanding cells in the body^19^ and they utilize glucose in the pentose phosphate shunt to produce intermediates required for outer segment renewal, and for adenosine triphosphate (ATP) production. The latter, though significant, is known to mainly maintain the dark current in photoreceptors. If the ATP level is too low, an energetic crisis that initiates cell death can occur but, under normal circumstances, ATP can be assumed constant. In these retina cells the metabolism of glucose through aerobic glycolysis is favored over oxidative phosphorylation and the tricarboxylic acid cycle (TCA); approximately 80–90% of glucose is converted to lactate^20^ in adult photoreceptors, even when oxygen is present. This phenomenon is well identified in cancer and other proliferating cells and is part of many photoreceptor-related investigations^16,20–24^. The produced lactate is then transported to the RPE and into the choroidal circulation^16^. When glucose is scarce, the expression of the glycolysis enzyme hexokinase-2 (HK2) increases^25^. HK2 helps photoreceptors retain glucose for glycolysis and consequently divert the subsequent metabolic product dihydroxyacetone phosphate (DHAP) to the Kennedy pathway, responsible of forming phospholipids and helping maintain the regeneration of photoreceptor outer segments^25–28^.

The RPE and the photoreceptors are metabolically coupled and act as a functional unit. The RPE oxidizes fatty acids from ingested outer segments and lactate produced through aerobic glycolysis in the outer retina to support its metabolic demands. This allows glucose from the choroidal circulation to be spared for the outer retina^28^. In healthy conditions, the RPE consumes more lactate than the photoreceptors, and the photoreceptors consume more glucose than the RPE^29^. The rods photoreceptors help the cones photoreceptors accelerate the uptake of glucose via the rod-derived cone viability factor (RdCVF). This symbiotic relationship maintains metabolic homeostasis, and genetic and environmental perturbation of either the RPE or photoreceptor cells can cause perturbations in nutrient flux leading to atrophy of the RPE and degeneration of photoreceptors. For example, if for any reason glucose uptake in insufficient in the retina, the lactate production by the photoreceptors for RPE metabolism decreases. In this case, the RPE retains the available glucose as a substrate for its own metabolism rather than transporting it to the photoreceptors^16,30^. Glucose can be metabolized by the RPE if lactate production by the outer retina is reduced or lactate transporters are inhibited. On the other hand, under glucose scarcity, the metabolites produced from the outer segments’ fatty acids can be used to fuel the photoreceptors instead of the RPE^16,30–32^; this is known to happen in patients with retinitis pigmentosa^33^. However, this seeming competition might in fact be the ecosystem’s defense mechanism to preserve its most critical functions when under stress.

GLUT1 is found on both the apical and basolateral membranes of the RPE^34,35^ to facilitate transepithelial transport of glucose from choroidal vessels to the outer neural retina^28^. RdCVF binds to basigin-1 (Bsg1) and and stabilizes GLUT1 in the plasma membrane of photoreceptors to enhance glucose uptake. The GLUT1 deficiency syndrome (GDS) is an autosomal dominat disease caused by mutations in the solute carrier family 1 member 1 (SLC1A1) gene and is associated with many disorders including epilepsy, body movement disorders and speech impairments^36,37^. In Pascual et al.^38^, several non eye-specific GLUT1 deficiency disorders are reviewed. However, GDS has not been found to impact vision in humans or in mice that are heterozygous for the solute carrier family 2 member 1 (SLC2A1) gene. In fact, results of several experimental works suggest that GLUT1 inhibition can suppress diabetic retinopathy by decreasing retinal glucose and, thus, GLUT1 is often targeted by diabetic retinopathy drugs^39^. Nevertheless, as demonstrated in Henry et al.^39^ by means of a human case study, GLUT1 inhibition can be detrimental for retinal activity and vision because of its contribution to photoreceptor degeneration. Indeed, experiments in mice have shown that if GLUT1 expression falls below a certain critical threshold then photoreceptor outer segments (OS) renewal and photoreceptor cell survival are negatively affected; the impact was found to be greater for rods (reflected by a 50% reduction in rod photoreceptor OS length and a 50% rod cell death) than for cones (reflected by a 10% reduction in cone photoreceptor OS length and a 17% reduction in cone cell number^28^). Still, the cones are also ultimately affected by GLUT1 deprivation due to their dependence on RdCVF, synthesized by rods^22^. In Retinitis Pigmentosa, photoreceptors upregulate GLUT1 and HK2^40,41^, which indicates that these cells are attempting to metabolize more glucose through aerobic glycolysis. Investigating the mechanisms that promote or hinder rod survival is thus of great importance to understand the progression of this and other diseases related to both types of photoreceptors’ death that lead to irreparable blindness, such as age-related macular degeneration. Insights in this area will pave the way towards helping design therapeutic treatments that might stop, reverse and even prevent such conditions. On the other hand, it has been observed that the deletion of GLUT1 does not affect the RPE’s differentiation, functions, or the polarity of monocarboxylate transporter 1 (MCT1) and 3 (MCT3) -responsible of lactate transport- but that it significantly impacts the capacity of the RPE to transport glucose into the outer retina^28^.

In this work we study the physiological connections between the RPE and the rods in terms of metabolic pathways, metabolite exchange, and the regulating mechanisms that govern them. We propose a mathematical model of the RPE-rod unit focusing on glucose and lactate transport where a feedback loop between the RPE and the rods is in place, and the delicate balance between the different metabolic pathways, necessary for cell homeostasis, is kept by a set of regulating mechanisms. We define feedback interconnections through which glucose and lactate are interchanged, and characterize their regulation by the abundance or lack of other metabolites, enzymes or proteins. In order to validate the accuracy of our model, we run computational simulations and compare our numerical results of metabolite concentrations in a healthy cell to those reported in Kanow et al.^42^. The immediate objective of the described model is to analyze the impact of GLUT1 modulation in the different metabolic steps of RPE and rods in order to draw conclusions on their robustness and ability to adjust or adapt to disturbances. Through computational research we identify dangerous conditions before cell degeneration occurs, as well as potential mechanisms that might contribute to the development of diseased states or cell death. To this end, we run simulations of our model with similar conditions of GLUT1 concentration to the ones reported in Swarup et al.^28^ and make sure that our model accurately reproduces the experimental results. We then test conditions beyond the experimentally examined ones, obtaining a large set of numerical data.

This is the first model of its type to consider the interactions between the RPE and the rods. It is also the first to allow modifications to its parameters to represent different states of health or disease. We have aimed here to include the most significant key components in order to optimize the model construction and its corresponding mathematical calculations. The ultimate goal of this model is not to replace wet experiments, but to inform and guide them. In turn, the data produced by these future experiments can be then used to better tune the model, thus completing a cycle that, we believe, can advance the field. We expect this work to be the starting point in building a complete but practical computational model of the lactate-mediated metabolic exchange in the retina.

The remainder of this manuscript is organized as follows: “Mathematical model” section describes the proposed model that defines the dynamics of the rods and the RPE. First, a high-level block diagram that sketches the structure of the considered connections and internal pathways of the two cells is presented. Then, the key metabolites that integrate the metabolic pathways are organized in the form of a network, which also includes the regulating functions. Last, a set of differential equations that constitute the mathematical model is presented. “Healthy conditions” section reveals the numerical results of the dynamical system (represented by nonlinear equations), and explains how our model is validated using experimental data. “[GLUT1] modulation” section presents the motivation and predictive capabilities of this work by illustrating the effect that modulating GLUT1 has in the metabolite concentrations of the rods and the RPE. Finally, “Conclusions” section summarizes the results, presents some perspectives and proposes directions for future work.

## Results

### Mathematical model

#### Metabolic pathways and regulating mechanisms

**Figure 1:**
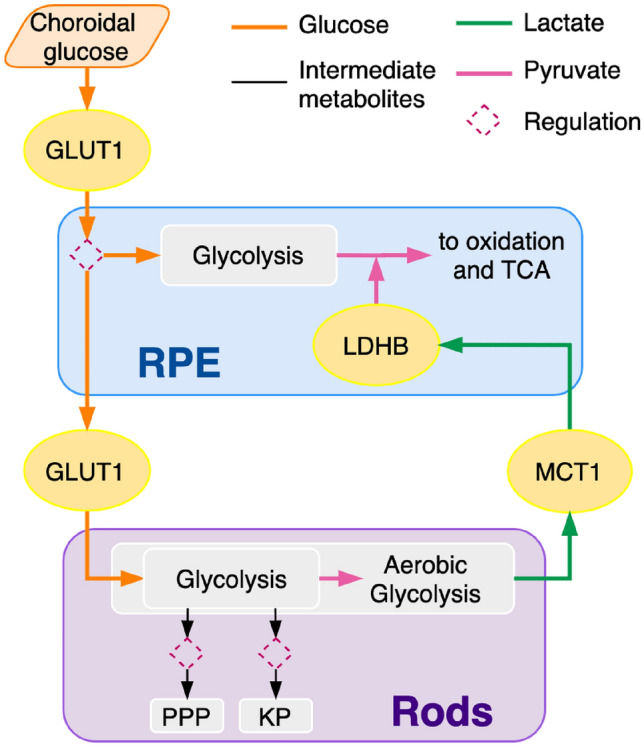
Diagram of key metabolic pathways (glycolysis, aerobic glycolysis, penthose phosphate pathway (PPP), and Kennedy pathway (KP)), transporters (GLUT1, MCT1), enzymes (lactate dehydrogenase B (LDHB)), and feedback metabolite flow between the RPE and the rods considered in our model. The metabolic pathway blocks represent several enzymatic reactions, and each reaction uses a specific metabolite as substrate to produce another metabolite. The RPE transports glucose from the choroid into the photoreceptors and keeps only a small portion to be converted into pyruvate through glycolysis. The transport is enabled by GLUT1. Once in the photoreceptors, glucose enters the glycolysis pathway from which intermediate metabolites are directed to the PPP and KP. Pyruvate, the end product of glycolysis, is reduced into lactate by aerobic glycolysis in the rods. Lactate is cleared from the rods, and transported to the interphotoreceptor matrix by MCT1, which also transports it into the RPE. Inside the RPE, lactate is converted to pyruvate by LDHB to be oxidized and processed by the tricarboxylic acid cycle, also known as the Krebs cycle.

To explore the key metabolic mechanisms in the RPE and rods we consider these systems in a vacuum with a minimal number of elements. In order to optimize the feasibility of the mathematical analysis we have opted to limit the amount of components to only those of the most significant contributions as per the available information. Figure 1 shows a diagram of the model that we propose. This diagram describes the metabolic flow, pathways, and feedback mechanism between the RPE and the rods considered in our work, isolating key elements of lactate and glucose exchange. The four pathways that we consider in this work are: glycolysis, aerobic glycolysis, penthose phosphate pathway (PPP) and Kennedy pathway (KP). The inclusion of these particular pathways will allow us to obtain quantitative data of the influence of the regulating mechanisms on each other and on the overall health of the cells. The modular structure of the model facilitates the further addition of other components that are not included in this first approach in its kind.

##### RPE

Glucose is transported from the choroid into the RPE by GLUT1, on its basal surface. On its apical surface, glucose is transported into the outer retina also by GLUT1. It has been found that the RPE directs most of the available glucose to the outer retina where it is metabolized through aerobic glycolysis, producing large amounts of lactate^29^. The MCT1 in the apical membrane facilitates the uptake of lactate from the subretinal space where it is converted into pyruvate by lactate dehydrogenase B (LDHB) to be oxidized and in the TCA cycle^42^ (see Fig. 1). Excess lactate is transported out of the RPE to the choroidal venules via MCT3 found in the basolateral membrane of the cells. When the RPE cannot get sufficient lactate for metabolism, it metabolizes glucose coming from the choroid (for use in glycolysis), rather than sparing it for the photoreceptors. This eventually diminishes the glucose in the rods. This follows the findings in Léveillard^16^ which establish that the direction of the glucose flux depends on the difference in lactate concentration between the interphotoreceptor matrix and the cytoplasm of the RPE cell. In the same work it is stated that a deficit in lactate clearance at the basal side of the RPE will trigger a decrease in transport on the apical side, resulting in an increase of lactate concentration in the inter-photoreceptor matrix (the space between photoreceptors) and reversing the polarity of lactate transport. It is also known that the presence of enough lactate to fulfill the RPE’s pyruvate needs blocks glycolysis in the RPE^42^ which, again, suggests that if lactate is insufficient in the retina, the RPE’s glycolysis process is unblocked and therefore transepithelial transport of glucose from the choroidal circulation to the photoreceptors is decreased. This indicates the presence of a lactate-dependent glucose regulation mechanism in the RPE, controlling the amount of glucose directed to the photoreceptors versus the amount uptaken by the RPE. In our model we only consider the RPE and the rods and focus on the interplay between glucose transport to the rods and lactate from these photoreceptors fueling the RPE metabolic process. Since we do not explicitly model lactate in the RPE we use its pyruvate concentration as a proxy that, together with the external lactate from the rods, modulates the changes in glucose transport to the rods in response reduction in the RPE’s metabolic fuel.

##### Rods

Four metabolic pathways will be considered in the rods’ part of our mathematical model (see Fig. 1): Glycolysis, pentose phosphate pathway (PPP), Kennedy pathway (KP) and Aerobic Glycolysis. Glucose entry is enabled by GLUT1 and is incorporated to the glycolysis pathway. Intermediate products of glycolysis are used to feed the PPP and the KP. The PPP produces nicotinamide adenine dinucleotide phosphate (NADPH) which reduces oxidized glutathione to reduced glutathione, that then neutralizes the toxic effects of reactive oxidative species (ROS)^23^, helping the cells cope with oxidative stress. The KP has two branches: DP-choline and CDP-ethanolamine. These are the predominant pathways responsible for the synthesis of the most abundant phospholipids, phosphatidylcholine and phosphatidylethanolamine in mammalian membranes^43^. It regulates the balance between phospholipids and neutral lipids^44^, and produces glyceraldehyde 3-phosphate (G3P), (the precursor of the glycerol backbone of phospholipids), to promote the OS renewal process. In the KP, fatty acids are combined with glycerol (produced from the G3P) to form the phospholipids which is necessary for membrane synthesis^16^. Note that this part of our model represents a whole rod, unlike previous models that describe the functions of photoreceptors outer segments only. It has been established that the central metabolic role of photoreceptors in retinal energy metabolism is to convert glucose to lactate, and that lactate is distributed to the RPE to be used as fuel^42^. This is done through aerobic glycolysis^18^. It has been demonstrated experimentally that aerobic glycolysis is also necessary for replenishing 10% of the OS that are shed each day by the photoreceptors^45^. The large amounts of lactate produced is cleared from the rods by MCT1, which also enables the RPE to absorb this metabolite for its conversion into pyruvate, to be utilized in its TCA for energy production^46^. If lactate cannot be cleared from the photoreceptors and transported into the extracellular space, the concentration of internal lactate increases, provoking a shift in the gradient that leads to the conversion of lactate back into pyruvate using LDHB. This pyruvate then goes to the mitochondria to facilitate in ATP production. Pyruvate leakage from the mitochondrial respiratory chain results in the accumulation of reactive oxidative species which contributes to photoreceptor degeneration^47^. Under healthy conditions, glycolysis in photoreceptors favors lactate formation rather than lactate utilization. Photoreceptors also divert some of the pyruvate that they produce to oxidative phosphorylation (OXPHOS) for the TCA cycle instead of using it for lactate production, but the amount is generally estimated to be between 5 and 20% only for experimental animal models^48,49^ and thus it is not considered in this model. The glucose uptake, lactate clearance, and intermediate metabolite redirection operations depend on a delicately balanced homeostasis, which suggests the existence of various regulating mechanisms to ensure it.

#### Network

**Figure 2:**
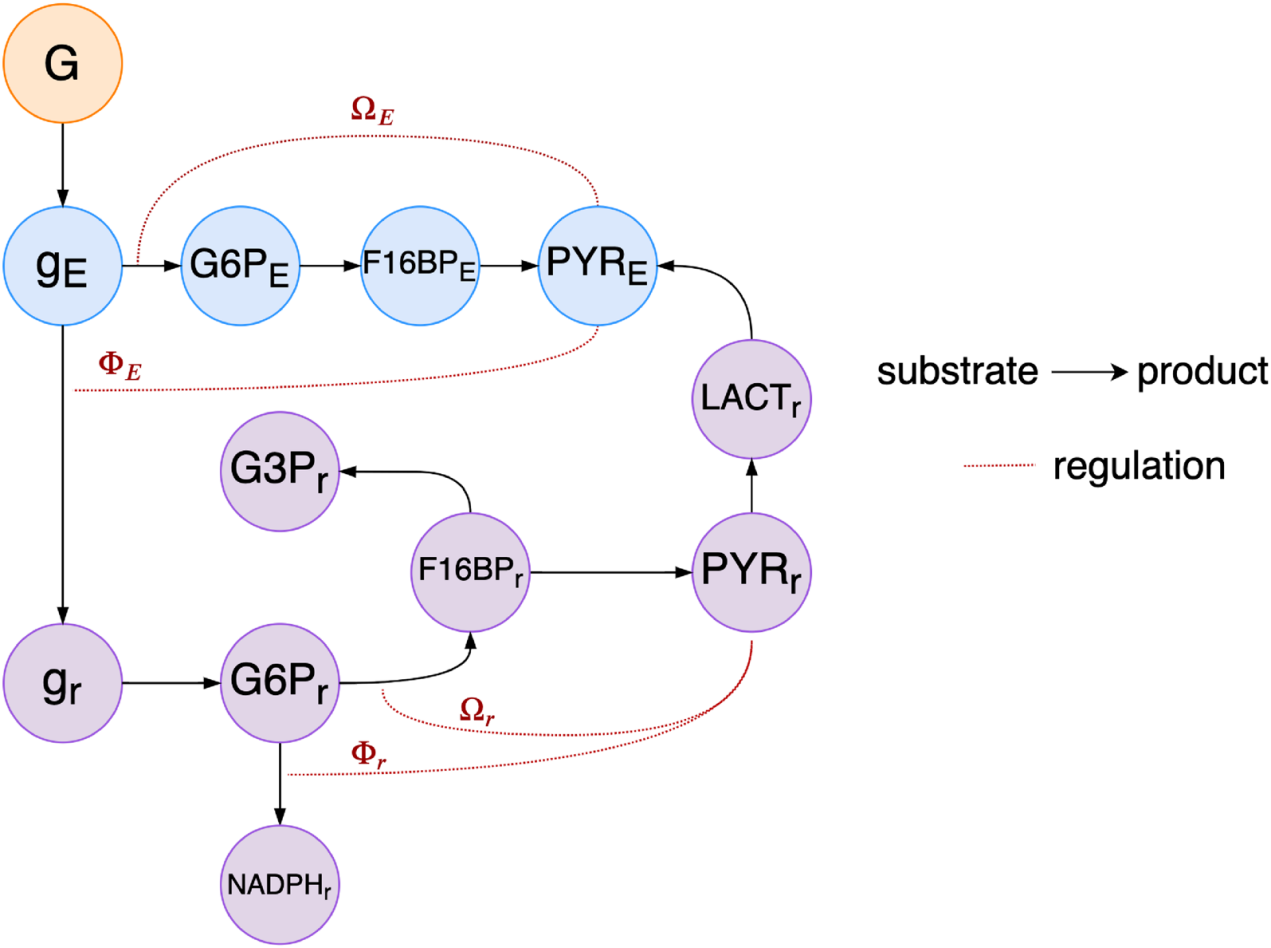
Network structure of the metabolite interactions in the rods and the RPE considered in our proposed mathematical model. Every node in the network represents the abundance of a specific metabolite. Blue nodes correspond to the metabolites in the RPE, denoted by the subscript *E*, and purple nodes correspond to the metabolites in the rods, denoted by the subscript *r*. The orange node represents the extracellular glucose. The enzymatic reactions by which metabolites are used and produced are represented by links: a link $x\rightarrow y$ indicates that the concentration of metabolite *y* directly depends on the concentration of metabolite *x*, in a substrate $\rightarrow$ product manner. A link $x {--}\downarrow$ indicates that the corresponding reaction is regulated by a mechanism dependent of metabolite *x*. The abbreviations used for the metabolites are: [g$_z$](*t*) for glucose inside the cell, [G6P$_z$](*t*) for glucose 6-phosphate, [F16BP$_z$](*t*) for fructose 1,6-bisphosphate, [NADPH$_z$](*t*) for nicotinamide adenine dinucleotide phosphate, [PYR$_z$](*t*) for pyruvate, [LACT$_z$](*t*) for lactate, and [G3P$_z$](*t*) for glyceraldehyde 3-phosphate.

Each pathway in Fig. 1 encompasses several metabolic steps that transform glucose into various intermediate products, and that are governed by specific enzymatic reactions. Figure 2 portrays the metabolic steps considered in our mathematical model, as well as the interaction between metabolites and the mechanisms that regulate such interactions, in the form of a network. Every node represents the abundance of a specific metabolite: blue nodes correspond to the metabolites in the RPE, denoted by the subscript *E*, and purple nodes correspond to the metabolites in the rods, denoted by the subscript *r*. The orange node represents the extracellular glucose. The enzymatic reactions by which metabolites are used and produced are represented by links: a link $x\rightarrow y$ means that the concentration of metabolite *y* directly depends on the concentration of metabolite *x*, in a substrate $\rightarrow$ product manner. A link $x{--} \downarrow$ means that the corresponding reaction is regulated by a mechanism dependent on metabolite *x*. The regulating functions of our system, denoted $\Omega _{E}$, $\Phi _{E}$, $\Omega _{r}$, and $\Phi _{r}$, are either of the activation or inhibition type, and they correspond to the regulating mechanisms previously described and in Fig. 2. These functions and their activator or inhibitory properties will be defined in “Mathematical model derivation” section. Every metabolite included in our model plays a role in either reflecting the health of the cell, or dictating the behavior of the regulating mechanisms. Other metabolic steps and pathways that exist in the rods and the RPE have been excluded from this mathematical model in order to keep a minimal amount of variables while getting enough significant quantitative information. This network will serve as a basis to define the mathematical equations that underlie the RPE-rod ecosystem, which will also be established in “Mathematical model derivation” section.

#### Mathematical model derivation

Through the rest of this section we will establish mathematical expressions that describe the dynamics of the model proposed in Figs. 1 and 2. In this work we use the notation defined below.

For a cell *z*, where $z=E$ represents the RPE and $z=r$ represents the rods (Fig. 2), the abundances of its metabolites at time *t* are represented by the following variables:

- $[\text{g}_z](t)$: glucose inside the cell,
- $$[\text{G6P}_z](t)$$: Glucose 6-phosphate,
- $$[\text{F16BP}_z](t)$$: Fructose 1,6-bisphosphate,
- $[\text{NADPH}_z](t)$: Nicotinamide adenine dinucleotide phosphate,
- $$[\text{PYR}_z](t)$$: Pyruvate,
- $$[\text{LACT}_z](t)$$: Lactate, and
- $$[\text{G3P}_z](t)$$: Glyceraldehyde 3-phosphate.

For simplicity, the argument (*t*) will be dropped in the rest of this paper. The metabolites pertaining to the RPE’s metabolic processes will be denoted as $z=E$, and those pertaining to the rods’ metabolic processes will be denoted as $z=r$. The term $\frac{d[\text{y}]}{dt}$ represents the rate of change of metabolite [y]. The term $\nu _{{\text{y}}}$ represents the kinetic rate functions of metabolite [y] . $V_{\mathrm{max}_{\mathrm{[y]}}}$ is the maximum transport rate of metabolite [y], and $K_{m_{\mathrm{[y]}}}$ is the substrate concentration that gives half the maximum of the transport rate of metabolite [y].

Building from the mathematical model and framework in Camacho et al.^50^, and adapting similar reactions and steps, we develop our model. We consider the change in the concentration of substrate with respect to time to be equal to the reaction rate for that concentration of substrate minus the reaction rate of the concentration of the product resulting from catabolizing that substrate with an enzyme. For example, the change in the concentration of glucose in the RPE, $\frac{d[\text{g}_E]}{dt}$, is given by its own kinetic rate function $\nu _{\mathrm{g}_E}$, minus the kinetic rates of the metabolites resulting from catabolizing [g$_E$], i.e., $\nu _{\mathrm{G6P}_E}$ and $\nu _{\mathrm{g}_r}$:

$$\begin{aligned} \frac{d[\text{g}_E]}{dt} =\nu _{\mathrm{g}_E} - \nu _{\mathrm{G6P}_E} - \nu _{\mathrm{g}_r}. \end{aligned}$$

Using the above expression and following the same method for every metabolite (node) in Fig. 2, we can readily establish the set of differential equations that describe the rate of production of every metabolite in our model as:

1 $$\begin{aligned} \frac{d[\text{g}_E]}{dt}&= \nu _{\mathrm{g}_E} - \nu _{\mathrm{G6P}_E} - \nu _{\mathrm{g}_r} \end{aligned}$$

2 $$\begin{aligned} \frac{d[\text{G6P}_E]}{dt}&=\nu _{\mathrm{G6P}_E} - \nu _{\mathrm{F16BP}_E} \end{aligned}$$

3 $$\begin{aligned} \frac{d[\text{F16BP}_E]}{dt}&=\nu _{\mathrm{F16BP}_E} - \nu _{\mathrm{PYR}_E} \end{aligned}$$

4 $$\begin{aligned} \frac{d[\text{PYR}_E]}{dt}&= \nu _{\mathrm{PYR}_E} + \nu _{\mathrm{PYR}_{rE}} - \psi _{\mathrm{PYR}_E}[\text{PYR}_E] \end{aligned}$$

5 $$\begin{aligned} \frac{d[\text{g}_r]}{dt}&=\nu _{\mathrm{g}_r} - \nu _{\mathrm{G6P}_r} \end{aligned}$$

6 $$\begin{aligned} \frac{d[\text{G6P}_r]}{dt}&=\nu _{\mathrm{G6P}_r} - \nu _{\mathrm{F16BP}_r} - \nu _{\mathrm{NADPH}_r} \end{aligned}$$

7 $$\begin{aligned} \frac{d[\text{NADPH}_r]}{dt}&=\nu _{\mathrm{NADPH}_r} - \psi _{\mathrm{NADPH}_r} \text{[NADPH]} \end{aligned}$$

8 $$\begin{aligned} \frac{d[\text{F16BP}_r]}{dt}&=\nu _{\mathrm{F16BP}_r} - \nu _{\mathrm{PYR}_r} - \nu _{\mathrm{G3P}_r} \end{aligned}$$

9 $$\begin{aligned} \frac{d[\text{G3P}_r]}{dt}&=\nu _{\mathrm{G3P}_r} - \psi _{\mathrm{G3P}_r} \text{R}_p [\text{G3P}_r] \end{aligned}$$

10 $$\begin{aligned} \frac{d[\text{PYR}_r]}{dt}&= \nu _{\mathrm{PYR}_r} - \nu _{\mathrm{LACT}_r} \end{aligned}$$

11 $$\begin{aligned} \frac{d[\text{LACT}_r]}{dt}&=\nu _{\mathrm{LACT}_r} -\psi _{\mathrm{LACT}_r} \text{[MCT1]} [\text{LACT}_r]. \end{aligned}$$

The Eqs. (4), (7), (9), and (11) have additional terms that are not kinetic rate functions and that represent the amount of the corresponding metabolite consumed by a particular pathway. Specifically, the terms $\psi _{\mathrm{PYR}_E},\psi _{\mathrm{NADPH}_r},\psi _{\mathrm{G3P}_r}$, and $\psi _{\mathrm{LACT}_r}$ represent the consumption or clearance by the RPE’s TCA, the rods’ PPP, the rods’ KP, and the rods’ MCT1-dependent lactate clearance process, respectively, and are approximated by constants. The metabolites whose dynamics include these consumption or clearance terms correspond to the *end nodes* in Fig. 2, i.e., those nodes do not have any outgoing links. The details of the remaining terms of the equations are discussed below.

The solutions to the differential Eqs. (1)–(11) represent the concentrations of metabolite at any time *t*. From Fig. 2 we see that the formation of every product depends on only one substrate, except for the pyruvate in the RPE, which has two arrows coming into it. This is reflected in the fact that every Eqs. (1)–(11) has only one positive term, except for (4) that has two. This information will be key to determining every metabolite’s kinetic rate functions below. The first step in our model is the incorporation (or production) of glucose inside the RPE, denoted by [g$_E$], which is the result of the binding of glucose outside the cell [G], which is the substrate, with the transporter GLUT1, forming a new complex. This new complex then releases glucose inside the cell as a product, preserving the transporter^51^. The reaction can be written as:

12 $$\begin{aligned} \text{[G]} + \text{[GLUT1]} \rightleftharpoons \text{[G/GLUT1]} \rightarrow [\text{g}_E] + \text{[GLUT1]}. \end{aligned}$$

We take the activation of glucose as an allosteric reaction, which means that there is a binding time requirement for the enzyme to catalyze the formation of the product. We model this kind of reaction as a functional response of Holling’s Type III, in accordance with previous works^11,50^, where the exponent applied to the variables in the numerator and denominator qualitatively portrays the reaching time and approximation to saturation of every reaction. Thus, the kinetic rate function $\nu _{\mathrm{g}_E}$ is given by

13 $$\begin{aligned} \nu _{\mathrm{g}_E} = \lambda _E \, [\text{GLUT1}] \, (\text{[G]} - [\text{g}_E]) \, \frac{V_{\mathrm{max}_{[\text{g}_E]}}\text{[G]}^2}{K_{{m}_{[\text{g}_E]}}^2+ [\text{G}]^{2}}, \end{aligned}$$

where $V_{\mathrm{max}_{[\text{g}_E]}}$ and $K_{m_{[\text{g}_E]}}$ are constant.

In these kind of reactions the enzyme is preserved, so the kinetic rate functions of the products are normally only a function of its substrate ([G] in this case). Since the transport of glucose follows the gradient of the external and internal glucose, and it is facilitated by GLUT1, the kinetic reaction is regulated by the term [GLUT1]([G]-[g$_E$]). The parameter [GLUT1] represents the transporter’s relative concentration with respect to a healthy retina. Its maximal allowed value is [GLUT1] = 1, which means that glucose absorption is facilitated in a normal manner due to a healthy concentration of the transporter. The minimum allowed value, [GLUT1] = 0, represents the case of GLUT1 knockout which means an absolute lack of transporter. In this case no glucose can be absorbed into the RPE. The term $(\text{[G]} - [\text{g}_E])$ indicates the direction of the glucose flow. It is important to note that in our model the abundance of the external choroidal glucose should always be kept larger than the internal RPE glucose, i.e., [G]>[g$_E$], so that the flow will be from the outside of the cell towards its inside, in accordance with the unidirectionallity of the second arrow in (12). Finally, $\lambda _E$ is a constant scaling factor.

The production of G6P in the RPE uses the glucose incorporated in the RPE as a substrate. The enzyme that enables this reaction is hexokinase 2 (HKII), and the reaction is written as

14 $$\begin{aligned}{}[\text{g}_E] + \text{[HKII]} \rightleftharpoons [\text{g}_E/HKII] \rightarrow [\text{G6P}_E] + \text{[HKII]}. \end{aligned}$$

This step marks the initialization of glycolysis in the RPE. In our proposed model it is regulated by the function $\Omega _{E}$, which depends on the concentration of pyruvate in the RPE (see the link [g$_E$] $\rightarrow$ [G6P$_E$] in Fig. 2). The glycolysis process in the RPE can be blocked or activated depending on whether enough lactate can be absorbed to fulfill the RPE’s pyruvate requirements or not. We model the RPE’s pyruvate requirement with the constant [PYR$_E$*], and use the following function^50^ to model the RPE glycolysis regulation:

15 $$\begin{aligned} \Omega _{E}= \frac{[\text{PYR}_E*]^4}{[\text{PYR}_E*]^4+[\text{PYR}_E]^4}. \end{aligned}$$

**Figure 3:**
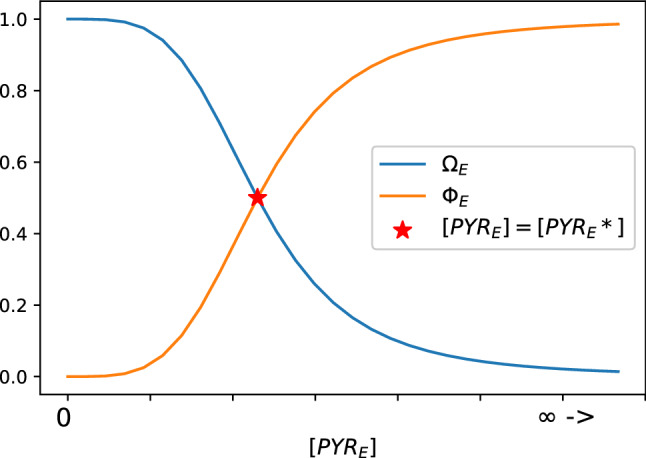
The regulating function $\Omega _{E} = \frac{[\text{PYR}_E*]^4}{[\text{PYR}_E*]^{4}+[\text{PYR}_E]^4}$ (blue line), and its complement $\Phi _{E} = (1-\Omega _{E})$ (orange line) take values between 0 and 1 depending on the concentration of [PYR$_E$] with respect to the constant [PYR$_E$*]. This parameter represents the healthy RPE’s requirement of pyruvate, and determines the proportion of glucose that the RPE keeps for its own glycolysis pathway, and the proportion that it is diverted to the rods. For low $\Omega _{E}$ (high $\Phi _{E}$), the RPE’s glucose utilization is blocked (and redirection of glucose to the photoreceptors is favored). Conversely, high $\Omega _{E}$ (low $\Phi _{E}$) favors the RPE’s retention of glucose, activating its glycolysis (and blocking the transport of glucose to the rods). Both lines intersect at precisely [PYR$_E$] = [PYR$_E$*] (red star).

Function (15) can take values between 0 and 1, and its curve with respect to the [PYR$_E$] concentration is shown in Fig. 3 (blue line). When the pyruvate concentration in the RPE is abundant with respect to [PYR$_E$*], i.e., [PYR$_E$] > [PYR$_E$*], the numerical value of $\Omega _{E}$ is low. This means that as long as there is enough [PYR$_E$], most of the glucose will be redirected to the rods and very little will be utilized by the RPE for glycolysis. If [PYR$_E$] becomes scarce, the value of $\Omega _{E}$ increases, favoring glucose uptake in the RPE, activating its glycolysis. We use the power 4 to approximate a step function without introducing the complication that a discontinuous function or higher powers would add. As a consequence of low levels of [PYR$_E$], the glucose available for intake by the rods will be reduced. From the reaction (14), and function (15), we define the kinetic rate function as

16 $$\begin{aligned} \nu _{\mathrm{G6P}_E} = \frac{V_{\mathrm{max}_{[\text{G6P}_E]}}[\text{g}_E]^{2}}{K_{m_{[\text{G6P}_E]}}^{2} +[\text{g}_E]^{2}}\,\Omega _{E}. \end{aligned}$$

Our model is designed to account for knock-out type experiments where a certain gene is disrupted or eliminated from specimens. The extreme case of a gene’s total deletion might lead to the total absence of a certain metabolite. Considering the case when the reserve of [PYR$_E$] is exhausted, i.e. [PYR$_E$]$\approx 0$, the regulating function (15) reaches its extreme value of $\Omega _{E} \approx 1$, activating glycolysis in the RPE at its maximum capacity. On the other hand, if [PYR$_E$] accumulates in extremely large quantities, the regulating function (15) becomes $\Omega _{E} \approx 0$, blocking the RPE’s glycolysis with $\nu _{\mathrm{G6P}_E} \approx 0$.

In our model we assume that any intermediate steps between the production of the G6P and F16BP in the RPE always happen once glycolysis has been initiated. For the remaining metabolic steps in the RPE we will skip writing the enzymatic reactions and we will focus on defining the kinetic rate functions that explicitly appear in our differential equations. The kinetic rate function that governs the production of F16BP using G6P as a substrate is given by

17 $$\begin{aligned} \nu _{\mathrm{F16BP}_E} = \frac{V_{\mathrm{max}_{[\text{F16BP}_E]}}[\text{G6P}_E]^{2}}{K_{m_{[\text{F16BP}_E]}}^{2}+[\text{G6P}_E]^{2}}. \end{aligned}$$

In our model, pyruvate is considered the end product of the RPE’s glycolysis. Any intermediate steps between the production of F16BP and the release of pyruvate are assumed to always happen and thus we model the pyruvate’s kinetic rate function using F16BP as a substrate as:

18 $$\begin{aligned} \nu _{\mathrm{PYR}_E} =\frac{V_{\mathrm{max}_{[\text{PYR}_E]}}[\text{F16BP}_E]^{2}}{K_{m_{ [\text{PYR}_E]}}^{2}+[\text{F16BP}_E]^{2}}. \end{aligned}$$

Note that this reaction is different from the one that converts the external lactate that the RPE receives into pyruvate, for which we use ﻿$\nu _{\mathrm{PYR}_{rE}}$ in (4), and that will be defined later.

The incorporation of glucose inside the rods is the result of the binding of the glucose outside the cell with [GLUT1]. In this case, the glucose outside the cell is represented here by [g$_E$] (the glucose that passes through the RPE), and its passage is regulated by the function $\Phi _E$, defined below and as illustrated by the link [g$_E$] $\rightarrow$ [g$_r$] in Fig. 2. The corresponding reaction can then be expressed as

19 $$\begin{aligned}{}[\text{g}_E] + \text{[GLUT1]} \rightleftharpoons [\text{g}_E/GLUT1] \rightarrow [\text{g}_r] + \text{[GLUT1].} \end{aligned}$$

The kinetic rate function of the rods’ glucose intake is modeled as

20 $$\begin{aligned} \nu _{\mathrm{g}_r} = \lambda _r\, \text{[GLUT1]}\, ([\text{g}_E] - [\text{g}_r])\, \frac{V_{\mathrm{max}_{[\text{g}_r]}} [\text{g}_E]^{2}}{\left( \frac{K_{m_{ [\text{g}_r]}}}{\Phi _{E}}\right) ^2+ [\text{g}_E]^{2}} \end{aligned}$$

where, in a similar way as in Eq. (13), we explicitly include the parameter [GLUT1] to represent the transporter’s relative concentration, and $\lambda _r$ is a constant scaling factor. In order to accurately represent the direction of glucose flow from the RPE to the rods, the term $([\text{g}_E] - [\text{g}_r])$ must be kept positive. The regulating function $\Phi _E$ is between 0 and 1 and acts to inhibit the catalytic reaction of the glucose activation in the rods when there is not enough pyruvate in the RPE and is defined as the complement of function $\Omega _E$, that is,

21 $$\begin{aligned} \Phi _E = 1-\Omega _{E}. \end{aligned}$$

In other words, when $\Phi _E <1$, the catalytic reaction in (20) will get inhibited and the substrate concentration giving half maximal velocity of the reaction catalyzed by the allosteric enzyme, defined as $\left( \frac{K_{m_{[\text{g}_r]}}}{\Phi _{E}}\right)$, is increased.

Function (21) can take values between 1 and 0, and depends on the value of [PYR$_E$] with respect to a constant [PYR$_E$*] (see Fig. 3). If pyruvate is scarce in the RPE, i.e., [PYR$_E$]<[PYR$_E$*], the numerical value of $\Phi _E$ is very low, thus increasing the inhibition of the catalytic reaction in (20) and lowering the rods’ intake of glucose. If there is sufficient pyruvate to meet the RPE’s requirements, i.e., [PYR$_E$]>[PYR$_E$*], the numerical value of $\Phi _E$ is high, thus lessening the inhibition of the reaction in (20) and increasing the rods’ intake of glucose.

The rods initiate their glycolysis process after glucose intake. In our model the rods’ glycolysis consists of the same three steps as the RPE’s glycolysis: the conversion of glucose into G6P, the conversion of G6P into F16BP and the conversion of F16BP into pyruvate. Again, we assume that any intermediate steps between these three always happen. The reactions that govern these steps are assumed to be identical to the corresponding ones in the RPE’s glycolysis, except for their regulating terms. The kinetic rate function of the rods’ first glycolytic step, which uses glucose as a substrate to produce G6P, is then modeled by:

22 $$\begin{aligned} \nu _{\mathrm{G6P}_r} = \frac{V_{\mathrm{max}_{[\text{G6P}_r]}}[\text{g}_r]^{2}}{K_{m_{ [\text{G6P}_r]}}^{2}+[\text{g}_r]^{2}}. \end{aligned}$$

One major difference between the RPE’s and the rods’ metabolic processes in our model is that while it is assumed that all the glucose that starts down the glycolytic pathway in the RPE is used to produce pyruvate, in the rods we also consider the diversion of glycolysis intermediate products to the PPP and the KP (see the rods’ glycolysis block in Fig. 1).

Following Camacho et al.^50^, we model the diversion of glucose into these pathways. The regulating function $\Phi _{r}$ can take values between 1 and 0, and determines the proportion of the rods’ G6P that will be directed to PPP, enabling the production of NADPH. Its complement, $\Omega _r = 1-\Phi _r$, determines the proportion of the rods’ G6P that will continue to the next step of glycolysis, which produces F16BP. In Camacho et al.^50^, [PEP*] is defined to be the critical amount of phosphoenol pyruvate (PEP) that grants the diversion of glucose into the PPP or down the glycolysis path. PEP is a metabolite produced in an intermediate step, right before the production of pyruvate, and is not explicit in our equations; therefore, we use [PYR]$_r$ as a proxy for it. Accumulation of PEP in the rods results in the inhibition of triose phosphate isomerase, which is necessary for the diversion of G6P down the glycolysis pathway. In this case, the rerouting of G6P into the PPP is activated. This also interrupts diversion of glucose into KP, the subsequent catalytic reaction of G3P, and the production of phospholipids. The value of the regulating function $\Phi _r$ depends on the relation of [PEP*] to [PYR$_r$] and is defined as

23 $$\begin{aligned} \Phi _{r} = \dfrac{[\text{PYR}_r]^{4}}{\text{[PEP*]}^4+[\text{PYR}_r]^4}. \end{aligned}$$

The kinetic function rates of [F16BP$_r$] and [NADPH$_r$] are given by

24 $$\begin{aligned} \nu _{\mathrm{F16BP}_r} = \frac{V_{\mathrm{max}_{ [\text{F16BP}_r]}}[\text{G6P}_r]^{2}}{K_{m_{ [\text{F16BP}_r]}}^{2}+[\text{G6P}_r]^{2}} \, \Omega _{r}, \end{aligned}$$

and

25 $$\begin{aligned} \nu _{\mathrm{NADPH}_r} = \frac{V_{\mathrm{max}_{ [\text{NADPH}_r]}}[\text{G6P}_r]^{2}}{K_{m_{ [\text{NADPH}_r]}}^{2}+[\text{G6P}_r]^{2}}\,\Phi _{r}, \end{aligned}$$

respectively.

The function (23) and its complement $\Omega _r = 1-\Phi _r$ have analogous behavior to the functions $\Phi _E$ and $\Omega _E$ in (15) and (21). If there is not enough PEP to start PPP, i.e., [PYR$_r$]<[PEP*], the value of $\Phi _{r}$ will be low and the value of $\Omega _{r}$ will be high, which will favor glycolysis and inhibit the production of NADPH. In the opposite case, if PEP accumulation surpasses the critical value, i.e., [PEP*]<[PYR$_r$], the value of $\Phi _{r}$ will be high and the value of $\Omega _{r}$ will be low, which will favor the PPP and inhibit the production of F16BP and the rest of the glycolytic products. Looking at Fig. 3, if the horizontal axis is now the concentration of [PYR$_r$], that is a proxy for PEP, the orange line would represent $\Phi _r$ and the blue line would represent $\Omega _r$. The two lines meet at the red star, which in this case would represent the optimal PEP concentration, represented by [PYR$_r$] = [PEP*].

In our model, the last step in the glycolysis pathway of rods produces pyruvate from F16BP. Meanwhile, F16BP is also the substrate for the production of G3P in the KP. The diversion of F16BP towards the end of glycolysis and towards the KP is assumed to be regulated at a constant rate, represented by a constant $q_r\in (0,1)$, with a healthy value of $q_r=0.18$^50^. When $q_r<0.18$ glycolysis is favored and, when $q_r>0.18$, the KP is favored. The kinetic rate functions of [PYR$_r$] and [G3P$_r$] are given by

26 $$\begin{aligned} \nu _{\mathrm{PYR}_r} = \frac{V_{\mathrm{max}_{ [\text{PYR}_r]}}[\text{F16BP}_r]^{2}}{K_{m_{ [\text{PYR}_r]}}^{2}+[\text{F16BP}_r]^{2}}\, (1-q_r), \end{aligned}$$

and

27 $$\begin{aligned} \nu _{\mathrm{G3P}_r} =\frac{V_{\mathrm{max}_{ [\text{G3P}_r]}}[\text{F16BP}_r]^{2}}{K_{m_{ [\text{G3P}_r]}}^{2}+[\text{F16BP}_r]^{2}}\, q_r, \end{aligned}$$

respectively.

In most cells, if there is oxygen available, pyruvate is metabolized through the very efficient OXPHO (as is the case in the RPE); however, a distinctive characteristic of rods is that they opt to ferment pyruvate into lactate, even in the presence of oxygen. This process is known as aerobic glycolysis and is known to be a hallmark of cancer cells^16,20–23^. The transformation of the rods’ pyruvate into lactate is the last step of the rods’ metabolism in our model, and its kinetic rate function is

28 $$\begin{aligned} \nu _{\mathrm{LACT}_r} =\frac{V_{\mathrm{max}_{ [\text{LACT}_r]}}[\text{PYR}_r]^{2}}{K_{m_{ [\text{LACT}_r]}}^{2}+[\text{PYR}_r]^{2}} \, (1-\rho _r), \end{aligned}$$

where $\rho _r$ is a constant, with $0\le \rho _r\le 1$, and represents the proportion of ROS produced by leakage of the mitochondrial respiratory chain. In healthy conditions, very little leakage occurs.

The lactate produced by the rods is cleared towards the interphotoreceptor matrix and transported into the RPE by MCT1, closing the feedback loop between the two cells (see the green arrows in Fig. 1). We use the variable [LACT$_{Ex}$] to represent the amount of external lactate that is uptaken by the RPE. Note that this lactate is considered to exclusively come from the rods (cleared by MCT1) and not any other cell, i.e., lactate contributions to the RPE from any cell other than the rods are not included in our model. Because this step is not a chemical reaction, but the clearance of a metabolite from the rods regulated by a transporter protein, the amount of rod-supplied lactate that makes it into the RPE is calculated as an algebraic function of the abundance of MCT1, represented by the variable [MCT1], a constant scaling factor $\lambda _{\mathrm{ LACT}_{Ex}}$, and the concentration of lactate in the rods [LACT$_r$] as

$$\begin{aligned}{}[\text{LACT}_{Ex}]=\lambda _{{\text{LACT}}_{Ex}}\,\text{[MCT1]} [\text{LACT}_r]. \end{aligned}$$

It has been reported that an increased lactate abundance upregulates MCT1 expression^52,53^. As a first approximation we model the expression of MCT1 as a linear function of the abundance of lactate in the rods as

$$\begin{aligned} \text{[MCT1]} = c_{M1} \, [\text{LACT}_r] \end{aligned}$$

where $c_{M1}$ is a regulatory constant. In healthy conditions, MCT1 is abundant enough to allow a normal amount of lactate to be cleared out from the photoreceptors and transported into the RPE. This case is represented by the maximal value $c_{M1}= 1$. Any value of the parameter $c_{M1}< 1$ would mean that there is a shortage in MCT1 expression, decreasing the lactate that can be cleared out from the photoreceptors and transported into the RPE.The RPE uses the lactate lactate generated by the outer retina to produce pyruvate by means of LDHB. Recall that under healthy conditions the RPE acquires most of its lactate in this way, instead of by directly metabolizing glucose through glycolysis. Note that the minimum value $c_{M1}= 0$ represents a lack of MCT1 expression and, thus, a blockage of lactate clearance in the photoreceptors. Such blockage also prevents the lactate form being transported into the RPE, leading to a pyruvate shortage in it. This shortage activates its glycolysis, as modeled in (15). We use the variable $\nu _{\mathrm{PYR}_{rE}}$ to define the reaction by which the rod-supplied lactate is converted into pyruvate once inside the RPE, and to distinguish it from the lactate produced by the RPE’s glycolysis, as

29 $$\begin{aligned} \nu _{\mathrm{PYR}_{rE}} = \frac{V_{\mathrm{max}_{ [\text{PYR}_{rE}]}}[\text{LACT}_{Ex}]^{2}}{K_{m_{ [\text{PYR}_{rE}]}}^{2}+[\text{LACT}_{Ex}]^{2}}, \end{aligned}$$

so that the total growth of pyruvate concentration in the RPE, [PYR$_E$] in (4), is given by the sum of the one produced from rod-supplied lactate, and the one produced by the RPE’s glycolysis. We have now defined all the terms that form the system (1)–(11), which outlines the dynamics underlying the network in Fig. 2, and the feedback model in Fig. 1. In the next sections we will analyze the solutions of this dynamical system, which represent the abundance of every considered metabolite in time, both in healthy conditions and for some pathological cases. Our results will be validated by comparison with experimental data.

### Healthy conditions

**Figure 4:**
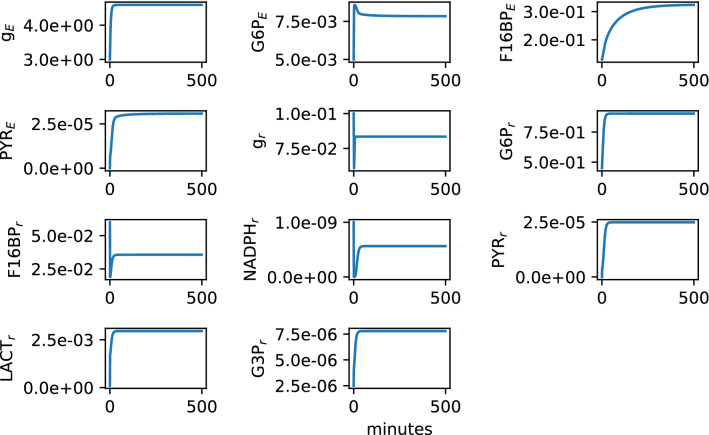
Metabolites’ steady state solutions. Here we show the time series resulting from numerically solving the system of differential Eqs. (1)–(11) for 500 min under healthy conditions, i.e., using the parameters and initial conditions of Table 1. Individual panels correspond to every node (metabolite) depicted in the network of Fig. 2, which is modeled by the differential Eqs. (1)–(11). Blue lines represent the concentration of the corresponding metabolite in time. After a short transient, or settling time, every metabolite reaches a feasible steady state. We refer to this steady state concentration as the *healthy concentration* or the *healthy case*, as it represents a qualitative baseline for analysing how the system responds to perturbations.

Table 1 contains the parameters that represent the healthy or nominal conditions of the dynamical system described in the previous section. Figure 4 shows the results of numerically solving the set of differential equations for the case when GLUT1 is abundant, i.e., [GLUT1] = 1, for 500 min. After a short transient, all the metabolite concentrations, defined by the trajectories of the solutions, reached a long term level (i.e., a steady state), which represents the healthy concentrations of each of the metabolites included in Fig. 2. Note that in order to maintain the the mathematical equations as simple as possible, our model includes only the key components and mechanisms to analyse the interplay between glucose and lactate consumption and transport between the rods and the RPE, assuming that some reactions always occur and clumping together some steps. For this reason, some of our chosen parameters may show discrepancies with respect to experimental measurements available in the literature, in which rates or concentrations were measured for specific isolated reactions or metabolites. Therefore, these parameters should not be interpreted as an exact quantitative values of what would be observed in the lab, but as a qualitative reference of the cells’ mechanisms and of how the functionality may change when perturbed. See SI 4 for a discussion on the model’s sensitivity to variations on the parameters.

**Table 1:** Parameters and initial conditions for the numerical simulations under healthy conditions.

Parameter	Value	Source	Parameter	Value	Source
G	5 mM	+^54^	$\lambda _E$	0.8 mM$^{-1}$	$\circ$
$V_{\mathrm{max}_{[\text{g}_E]}}$	1.2 mM min$^{-1}$	+^55^	$\lambda _{LACT_E}$	480 mM$^{-1}$	$\circ$
$K_{m_{[\text{g}_E]}}$	9 mM	^55^	$\lambda _r$	21.7E−3 mM$^{-1}$	$\circ$
$V_{\mathrm{max}_{[\text{G6P}_{E}]}}$	0.00738 mM min$^{-1}$	–	$\psi _{[\text{PYR}_E]}$	710 min$^{-1}$	$\circ$
$K_{m_{[\text{G6P}_{E}]}}$	0.002844 mM	–	$\psi _{[\text{NADPH}_r]}$	15E2 min$^{-1}$	+
$V_{\mathrm{max}_{[\text{g}_r]}}$	0.8 mM min$^{-1}$	^55^	$\psi _{[\text{LACT}_r]}$	96E2 min$^{-1}$	$\circ$
$K_{m_{[\text{g}_r]}}$	8 mM	^55^	$\psi _{[\text{G3P}_r]}$	0.02 min$^{-1}$	+
$V_{\mathrm{max}_{[\text{F16BP}_E]}}$	1.2285 mM min$^{-1}$	$\diamond$ +^56^	[GLUT1]	1	$\circ$
$K_{m_{[\text{F16BP}_E]}}$	0.1106 mM	–	$c_{M1}$	1	$\circ$
$V_{\mathrm{max}_{[\text{PYR}_E]}}$	0.00783 mM min$^{-1}$	–	$\delta _r$	13E8	$\circ$
$K_{m_{[\text{PYR}_E]}}$	0.17 mM	–	[PYR$_E$*]	4.6E−5 mM	$\circ$
$V_{\mathrm{max}_{[\text{PYR}_rE]}}$	14 mM min$^{-1}$	–	[PEP*]	1.65E−13 mM	$\circ$
$K_{m_{[\text{PYR}_rE]}}$	0.125 mM	*	$q_r$	0.18	+
$V_{\mathrm{max}_{[\text{F16BP}_r]}}$	1.365 mM min$^{-1}$	+^56^	$\rho _r$	1E−3	+^54^
$K_{m_{[\text{F16BP}_r]}}$	3.5 mM	+^56^	[g$_r$]$_0$	0.1 mM	–
$V_{\mathrm{max}_{[\text{G6P}_{r}]}}$	0.1845 mM min$^{-1}$	+^57^	[G6P$_r$]$_0$	0.45 mM	+
$K_{m_{[\text{G6P}_{r}]}}$	0.09 mM	+^54^	[F16BP$_r$]$_0$	0.06 mM	+
$V_{\mathrm{max}_{[\text{NADPH}_{r}]}}$	0.228 mM min$^{-1}$	+^58^	[NADPH$_r$]$_0$	0.1E−8 mM	–
$K_{m_{[\text{NADPH}_{r}]}}$	0.45 mM	+^58^	[PYR$_r$]$_0$	0 mM	^42^
$V_{\mathrm{max}_{[\text{PYR}_r]}}$	0.3915 mM min$^{-1}$	+^57^	[LACT$_r$]$_0$	1E−4 mM	+
$K_{m_{[\text{PYR}_r]}}$	0.06 mM	^57^	[G3P$_r$]$_0$	2.5E−6 mM	+
$V_{\mathrm{max}_{[\text{G3P}_r]}}$	0.15 mM min$^{-1}$	+^54^	[g$_E$]$_0$	3 mM	+
$K_{m_{[\text{G3P}_r]}}$	0.143 mM	+^54^	[G6P$_E$]$_0$	5E−3 mM	–
$V_{\mathrm{max}_{[\text{LACT}_r]}}$	0.14 mM min$^{-1}$	+	[F16BP$_E$]$_0$	0.13 mM	–
$K_{{m}_[\text{LACT}_r]}$	1.25E−5 mM	–	[PYR$_E$]$_0$	0 mM	^42^

The parameters marked as (+), are used exactly as they appear in Camacho et al.^50^, and the reference where they were originally reported is provided when applicable. The parameter marked as ($\diamond$) is within $\pm \,10{\%}$ of the value reported in the provided reference. The parameters marked as ($\circ$) are scaling constants added to this model and do not represent any real kinetic or concentration value in the cells. The parameters marked as (−) are estimated for this example by performing a systematic parameter sweep in which their values were changed by a small fraction. This sweep was iterated many times until the responses provided the best qualitative approximation to the available experimental data, as mentioned in “Model validation” section. The value of parameter $K_{m_{[\text{PYR}_{rE}]}}$ (marked with *) is equal to the value of parameter $K_{m_{\mathrm{[LACT]}}}$ in^50^ because the enzyme Lactate dehydrogenase b (LDHB) enables the transformation of pyruvate into lactate, and vice versa.

#### Model validation

**Figure 5:**
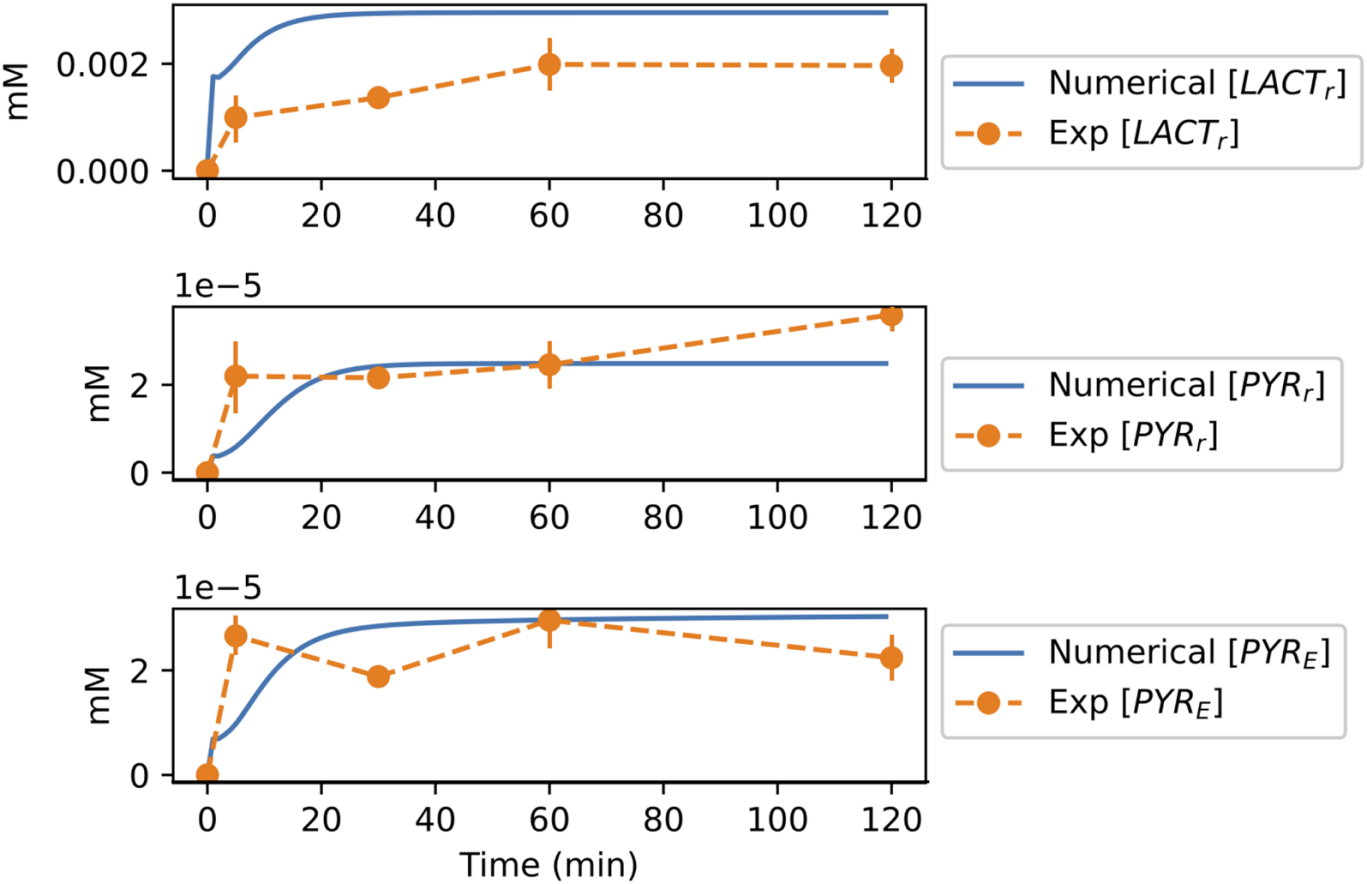
The concentrations obtained from running numerical simulations of system (1)–(11) for 120 min, compared to the mean of the experimental results in Kanow et al.^42^. Using a protein concentration of 20 mg/ml as reported in Du et al.^59^, we converted the mean of the experimental data (n = 3 for each time point)^42^ given in pM/$\mu$g, to mM/ml (orange markers) and compared our numerical results (blue lines) to the experimental data for lactate (top) and pyruvate (center) in the rods, and pyruvate in the RPE (bottom). In this figure the vertical orange lines represent the standard error.

To test the validity of the result given by our mathematical model, we compared the numerically obtained long term, i.e., steady state, concentration with experimental data. We used the experimental data in Kanow et al.^42^ to see how well it matched our model results. In this work, the incorporation of glucose to the retina and to the RPE were compared, and several metabolite concentrations in both cells were analyzed. In particular, they considered the lactate and pyruvate concentrations in both types of cells, at six different moments in time. First, we plotted the experimental data, performing a unit conversion assuming a protein concentration of 20 mg/ml (see SI 1 for details) as reported in Du et al.^59^ (the orange markers on Fig. 5 represent the mean values (n = 3 for each time point), and the vertical lines the standard error). For the first 15 min, approximately, the experimental metabolite concentrations show great variation, which is reduced after minute 30 for the lactate in the rods and the pyruvate in the RPE. We then plotted the numerical time series of lactate and pyruvate in the rods, and pyruvate in the RPE, using the basal values, i.e., initial conditions, and parameter values in Table 1 (continuous blue lines on Fig. 5). The transitory time and the qualitative approach to the equilibrium of our numerical simulations correspond to those of the available experimental data. After 60 min it can be expected that metabolite concentrations reach a relatively stable equilibrium, although this can be difficult to measure with great precision in the lab due to noise and instrument errors. For this reason, we focus on obtaining the closest qualitatively approximation to the experimental data rather than on accurately fitting every data point with our numerical simulations. By doing so, we obtain a baseline that represents a healthy case with which to compare the deviations that perturbations would induce.

### [GLUT1] modulation

The main motivation of this work is to analyse the effects of varying the concentration of GLUT1 in the rods and the RPE, and to investigate the role played by the regulating mechanisms in promoting glucose and lactate uptake and transport between the RPE and rods. It is widely known that glucose is the main energy source of the retina and that the photoreceptors metabolize it mainly through aerobic glycolysis, releasing lactate as a product. Also, it is known that the RPE’s main metabolic process is OXPHO, which needs pyruvate as a substrate, and that most of this pyruvate requirement does not come from its own glycolysis, but from the conversion of the lactate transported from the photoreceptors and other retina cells^29,46^. Therefore, the adequate regulation of the glucose and lactate flow within the retina is fundamental for maintaining sight. It has been experimentally demonstrated that GLUT1 is the main glucose transporter^36^, that it is found on both surfaces of the RPE^60^, and that the rods suffer from glucose deprivation induced by a decrease of GLUT1 expression^28^ ; however, the exact mechanisms that allow the RPE to maintain its basic functions when glucose is scarce, and the effects of varying GLUT1 beyond the experimentally tested concentrations are unknown. In this section we run numerical simulations of the mathematical model presented in “Mathematical model” section with different values of the parameter [GLUT1]. These values ranged from 1 (healthy concentration) to 0 (complete exhaustion), and we analyzed the steady state induced at every step. While knockout techniques allow one to experimentally test for a few different concentrations of GLUT1 (see Swarup et al.^28^ and discussion below), this numerical approach supports the examination of varying degrees of failure of the system with any desired resolution between them. This technique has been used to trace the progression of retinitis pigmentosa in Camacho et al.^9^ and to quantify the metabolic death kinetics of photoreceptors^10^.

**Figure 6:**
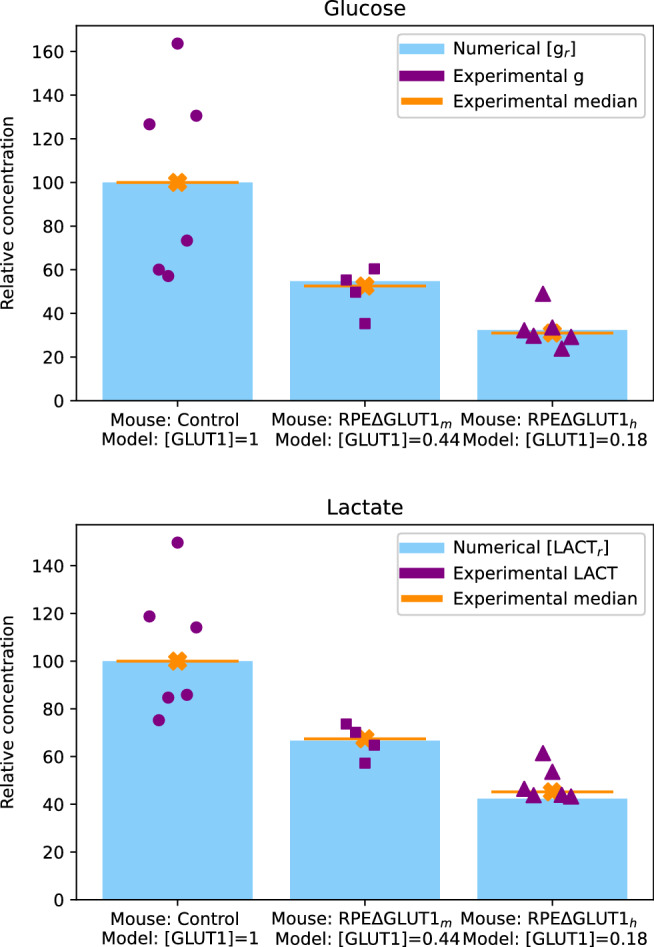
The relative metabolite concentrations obtained from running numerical simulations of system (1)–(11) match the experimental results of^28^. We iteratively ran our model with the parameters in Table 1 except for [GLUT1], which was changed by a small fraction every time. For each simulation we let our system run until all the metabolites had reached a steady state, and recorded the concentration values of glucose ([g$_r$]) and lactate ([LACT$_r$]) in the rods. We then calculated the relative concentrations of both metabolites with respect to the case when [GLUT1] = 1, i.e., we gave the control case a value of 100 and scaled the rest accordingly. Then, we looked for the [GLUT1] values for which the steady state most closely matched the data obtained in Swarup et al.^28^ for the metabolite intensity of RPE$\Delta$GLUT1$_m$, and RPE$\Delta$GLUT1$_h$ mice (expressing approximately 50% and less than 30% of the control GLUT1 concentration, respectively). The purple circles, squares and triangles mark the relative intensities of lactate and glucose in the the control, RPE$\Delta$GLUT1$_m$, and RPE$\Delta$GLUT1$_h$ mice, respectively, and the orange lines are the medians. Blue bars mark the numerical relative concentrations of [g$_r$] and [LACT$_r$] when [GLUT1] = 1 (left), [GLUT1] = 0.44 (center) and [GLUT1] = 0.18 (right), which are the closest match to the medians of the experimental data, and match the approximate GLUT1 expression in the mice. We found that the scaling of our numerical results match the corresponding scaled experimental results.

To test the validity of our model in predicting the effect of different levels of [GLUT1], we compared the results of our simulations with the experimental data reported in Swarup et al.^28^, obtained from using conditional knockout mice to study the impact on photoreceptors of reducing glucose transport across the RPE. In Swarup et al.^28^, the glucose and lactate intensities are measured in six specimens of control mice retinas, as well as in four specimens of medium GLUT1 knockout mice retinas (RPE$\Delta$GLUT1$_m$), which have approximately 50% RPE$\Delta$GLUT1 recombination, i.e., express approximately 50% of the control amount of GLUT1. The study also considers six specimens of high GLUT1 knockout mice retinas (RPE$\Delta$GLUT1$_h$), which have $>~70{\%}$ RPE$\Delta$GLUT1 recombination, i.e., express $<~30{\%}$ of the control amount of GLUT1. Since the available experimental data, obtained by mass spectrometry analysis of the specimens, was expressed in terms of metabolite intensities, and quantified with internal standards, we used relative concentrations in order to adequately compare it to our numerical data. To this end, we calculated the median of the glucose and lactate intensities found among the control specimens and we scaled every intensity measurement of each specimen with respect to that value (see SI 2 for details). The relative metabolites’ intensities are illustrated by the purple circles (control mice), squares (RPE$\Delta$GLUT1$_m$ mice), and triangles (RPE$\Delta$GLUT1$_h$ mice) in both panels of Fig. 6. The median of every group is shown by the orange horizontal lines. Note that the median of the control group, in both panels, has a value of 100, since the scaling was performed with respect to them. Next, we iteratively ran our model fixing all the parameters to the values in Table 1, except for [GLUT1] which was varied by a small amount for every run. We recorded every resulting steady state concentrations of [g$_r$] and [LACT$_r$], normalized to the case of [GLUT1] = 1 (see SI 2). Then, we looked for the set of numerical concentrations of both metabolites that most closely matched the medians of the experimental data (blue bars in Fig. 6). The [GLUT1] value that produced numerical results closest to the RPE$\Delta$GLUT1$_m$ mice median is 0.44, which matches the approximately 50% RPE$\Delta$GLUT1 recombination. Furthermore, [GLUT1] = 0.18 produced numerical concentrations of [g$_r$] and [LACT$_r$] that were closest to the median of the RPE$\Delta$GLUT1$_h$ mice which, correspondingly, express $<~30{\%}$ of the control amount of GLUT1. Note that the bars representing both cases of [GLUT1] = 1 in Fig. 6 indicate a value of 100 because the scaling was made with respect to them. This close match between the experimental data and the numerical concentrations prove that our model can accurately track changes in GLUT1.

#### Analyzing the impact of [GLUT1] modulation

To test the impact of modulating GLUT1, we ran new simulations decreasing the parameter [GLUT1] value from 1 to 0, in 100 equal steps, and analyzed the resulting metabolite levels represented by the trajectory of the solution curves. We initialized the simulations with the nominal value [GLUT1] = 1, let them run for 1500 min to ensure that a steady state had been reached, and recorded the resulting metabolite concentrations. These corresponded to the healthy state described in “Model validation” section. Then, we decreased the value of [GLUT1] by 0.01, let the simulations run for another 1500 min and recorded again the steady state. We repeated this procedure decreasing the [GLUT1] value at each step down to zero. To ensure that the changes in steady state concentration that we were recording were exclusively due to the [GLUT1] variation, we performed an initial conditions dependency test; we found that the resulting steady state concentration in every step of our experiment only depends only on the [GLUT1] concentration, and not on the initial metabolite concentration (see SI 3 for details). In order to analyze the change in concentration of all metabolites simultaneously, we normalized every metabolite’s concentrations, as the value of [GLUT1] varied, with respect to the steady state induced by [GLUT1] = 1. In other words, we made the nominal steady state values be 100 and scaled the rest accordingly (see SI 2 for details).

**Figure 7:**
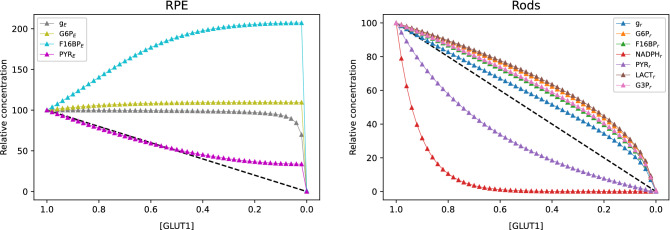
Relative steady state concentrations of all metabolites in the RPE and the rods as [GLUT1] decreases from 1 to 0, with all metabolites scaled to 100 at GLUT1 = 1 (representing the healthy case). Left panel: Metabolite concentrations in the RPE. The glucose concentration in the RPE (gray curve) stays close to its healthy value up until [GLUT1] has decreased by approximately 80% , i.e., [GLUT] = 0.2, likely due to the regulating mechanisms that block the glucose passage to the rods. There is a slight increase in the G6P and significant increase in the F16BP. Initially, the pyruvate concentration decreases at an almost constant rate because it highly depends on the rods’ contribution of lactate, which is lower with every step. However, when [GLUT1] decreases from approximately 0.45 down to 0.2, the rate at which pyruvate decreases and the rate at which F16BP increases both slow down. As [GLUT1] becomes even scarcer ([GLUT1] less than approximately 0.2), the glucose concentration decays rapidly. Right panel: Metabolite concentrations in the rods. The RPE’s regulating mechanisms block the passage of glucose to the rods, making its concentration decrease with every step. As [GLUT1] decreases, the production of aerobic glycolysis intermediate products is favored (slowest overall decrease). Among these are the production of G3P by the KP (essential for the rods’ OS renewal) and the production of lactate (that contributes to the RPE’s survival); the others are G6P and F16BP. Meanwhile, the production of NADPH (by the PPP, and the metabolite that undergoes the most rapid decrease) and pyruvate (that fuels the rods’ OXPHO) are the most affected by the response of the regulating mechanisms to the [GLUT1] decrease and the consequential glucose shortage. When [GLUT1] is less than approximately 0.2, the glucose directed to the rods is too small to maintain any of its functions, and all the metabolites concentrations rapidly go to zero.

The triangles in Fig. 7 represent the recorded relative steady state of every considered metabolite in our mathematical model, for each of the tested [GLUT1] values. The discontinuous black line in both panels illustrates the constant progression of [GLUT1] deprivation, from abundance ([GLUT1] = 1), to complete exhaustion ([GLUT1] = 0). The right panel of Fig. 7 shows that all the metabolite concentrations in the rods decrease as the [GLUT1] decreases, at different rates. This behavior is expected because as [GLUT1] decreases, less glucose, which is the source of all the other rods’ metabolites, can enter the system. This observation coincides with the progression of the [g$_r$] concentration, whose decrease is closest to the diagonal line. Note that this decrease is relatively slower (less steep slope) up until the value of [GLUT1] has dropped significantly (at [GLUT1] $\approx 0.3$). This phenomenon can be attributed to the regulating functions that allow less glucose to be directed towards the photoreceptors when the RPE experiences a shortage in its necessary nutrients. Note that towards the end of the experiment (when [GLUT1]$< 0.2$) the [g$_r$] concentration decays towards zero very quickly. This behavior is replicated by almost all the other metabolites in the rods, and will be interpreted later. The metabolite whose concentration is most rapidly affected is [NADPH$_r$]. This is shown by the slope of the red line in the right panel of Fig. 7 which is the steepest, indicating a faster rate of decrease. Reexamining the network of Fig. 2, note that the link [G6P$_r$]$\rightarrow$[NADPH$_r$] is regulated by the function $\Phi _r$ which, as defined in (10), regulates the metabolite flow between the rods’ glycolysis and PPP. The rapid decay of [NADPH$_r$] indicates that when a shortage of nutrients appears, the pathway that is more quickly disfavored is the PPP, thus favoring the continuation of glycolysis. In contrast, [LACT$_r$], [G6P$_r$], [G3P$_r$], and [F16BP$_r$] decay relatively slow in the rods as [GLUT1] decreases, as illustrated by the brown, orange, pink and green lines respectively, in the right panel of Fig. 7 that have less steep slopes for most of the experiment. These are glycolytic intermediates, obtained before and after the diversion of flow to the KP, as illustrated in Fig. 2. Recall that the production of G3P is essential for the rods’ OS renewal process and thus, is fundamental for their survival, as expressed in (20). This suggests that the KP was among the most favored pathways in the rods. By favoring the KP, the rods try to ensure their survival at the cost of disfavoring other metabolic pathways such as PPP and OXPHO. Although OXPHO is not explicitly included in our model, its obstruction is manifested by the rapid decay of [PYR$_r$] (purple line), which is its fuel substance. This is supported by the fact that pyruvate is the substrate for lactate production by aerobic glycolysis in the rods, so the quick reduction of [PYR$_r$] concentration and the contrasting slow decay of [LACT$_r$] (brown line) means that most of the obtained pyruvate is destined to feed aerobic glycolysis, thus still contributing to the RPE’s requirement of energy, at the cost of deterring its own OXPHO.

When [GLUT1]$<0.2$, most of the metabolites’ concentrations decrease very quickly, except for [NADPH$_r$] and [PYR$_r$] which are already very close to zero. In fact, at [GLUT1] = 0.2 the concentration of [G3P$_r$], [LACT$_r$], [G6P$_r$] and [F16BP$_r$] are around 50% of their nominal values. In contrast, the GLUT1 concentration is only 20% of what is considered healthy. Recall that when [GLUT1] $\ge 0.3$ the [g$_r$] slope is less steep than when [GLUT1] becomes smaller; we can interpret this behavior as the rods’ attempt for survival by trying to draw more glucose into the cell. This explains why the end products of KP and aerobic glycolysis can be still obtained at relatively high rates. However, as the GLUT1 deprivation continues, this effort ends up succumbing, drawing the concentration of all the metabolites to zero very rapidly, which leads to the cell’s inevitable death. This is known in the context of ecosystems as a critical transition, that is, a transition to a state from which the organism cannot recover anymore. In short, we can conclude from our observations that when GLUT1 becomes scarcer, the regulating mechanisms in the rods try to preserve certain functions at the expense of others. The function that is mostly protected is their OS renewal, powered by the KP, and the production of lactate by aerobic glycolysis which is then transported into the RPE. This can be interpreted as the rods choosing to mainly protect their own basic survival and to maintain their contribution to the RPE’s energetic process, at the cost of rapidly shutting down their own PPP and, at a slightly smaller pace, their OXPHO. This resistance of the rods against a perturbation such as GLUT1 deprivation can only be sustained up to a certain point (approximately when [GLUT1] = 0.2) after which all the rods functions are lost and the cell dies.

The left panel of Fig. 7 shows the RPE’s metabolite relative concentration variation as [GLUT1] changes, which displays some interesting behavior. GLUT1 is the facilitator of glucose absorption in the RPE but, even when its concentration is decreasing at a constant rate, the concentration of [g$_E$] stays very close to its nominal value for most of the experiment (gray line), only decreasing very quickly for [GLUT1]$\le$0.2 (identified as a critical value in the above paragraph). Under normal conditions, the RPE directs most of the glucose absorbed from the choroid to the photoreceptors, and this passage is regulated by function $\Phi _E$ (see the link [g$_E$]$\rightarrow$[G6P$_E$] in Fig. 2) which, as defined in (21), redirects this flow in the event that the RPE’s pyruvate requirements are not met (see Fig. 3). This requirement is mostly met by the photoreceptors’ contribution of lactate, transported into the RPE by MCT1, and converted to pyruvate by LDHB. We already discussed the decrease in the rods’ lactate concentration as soon as the GLUT1 deficiency appears, which decreases their contribution to the RPE’s pyruvate provoking a shortage. This induces the blocking of glucose redirection to the rods, favoring its retention in the RPE. Since more glucose is allowed to stay in the RPE, even when the transporter’s concentration decreases, the RPE is capable of maintaining a glucose concentration close to its healthy value. The next step in the RPE’s metabolic process is regulated by $\Omega _E$ (the complementary function of $\Phi _E$) which, by its definition in (15), activates the RPE’s glycolysis above its usual pace when pyruvate in the RPE is insufficient. This is the second consequence of the cutback in the rods’ lactate contribution and leads to the rapid consumption of glucose to produce G6P, reflected in the permanence of [G6P$_E$] close to its nominal value (olive line). G6P is converted into F16BP in the next step, which is also accelerated by the glycolysis activation of function $\Omega _E$. In fact, we can notice a large accumulation of [F16BP$_E$] in the RPE as [GLUT1] gets smaller (cyan line). We attribute this accumulation to the cell’s response to the lack of outside nutrients, which is more severe with every decrease in [GLUT1].

Now we look at the last step in the RPE’s metabolic pathway in our model, which involves the production of pyruvate from F16BP, and from the lactate received from the interphotoreceptor matrix. The sum of the concentrations resulting from these two reactions is represented by [PYR$_E$] (magenta line), which displays an almost constant rate of decrease as [GLUT1] declines, up until when [GLUT1] is approximately 0.45. After this point the slope of [PYR$_E$] flattens, maintaining a concentration that changes very little until the very last part of the experiment. This apparent stabilization of the [PYR$_E$] concentration can also be observed in the rise of the concentration of [F16BP$_E$], which we attributed above to the cell’s response to starvation. Interestingly, the same stabilization can be observed in the concentration of [G6P$_E$], as mentioned before. This phenomenon suggests that when the outside lactate begins to be scarce, the RPE prepares itself for survival without this contribution, switching to another state where it is capable of maintaining its functions autonomously. Remarkably, this mechanism seems to start very early in the progression of [GLUT1] deprivation. This explains how, although being highly dependent on the rods’ lactate under normal conditions, the RPE’s pyruvate can maintain a constant concentration while [GLUT1] concentration is below the identified critical level. However, this can only be maintained while enough glucose can enter the RPE to feed its glycolysis, which fails to happen by the end of the experiment. At this point [GLUT1] becomes so small that even with the passage of glucose to the rods completely blocked, as evidenced by the quick decrease of [g$_r$] analyzed before, the glucose kept in the RPE is insufficient to maintain its glycolysis. Then, the concentration of the RPE’s metabolites is drawn to zero very quickly, which we interpret as the death of the cell. In summary, in the case of a nutrient deprivation, the RPE’s activates mechanisms to preserve its functionality by decreasing the passage of glucose to the rods for its own use. The same mechanisms activate its glycolysis, progressively switching to a state where it can function independently from the rods. Nevertheless, as [GLUT1] becomes insufficient to bring in the minimal glucose required by the RPE, the cell will experience a critical transition to a state where all the metabolites concentrations go to zero, which leads to its death.

## Conclusions

We have presented here a model that synthesizes key metabolic functions in the rods and the RPE, the mechanisms that regulate them, and the metabolite feedback between the two types of cells. We illustrated the proposed model in the form of a block diagram and of a network, specifying the considered pathways, metabolites and regulating mechanisms. Based on the only existing mathematical model of the cones^50^, we built a mathematical representation of our model that enables the computational analysis of the concentration of key metabolites in the RPE-rods unit, under different conditions. We also selected a set of parameters that define the healthy state of both cells, according to various sources in the literature, and estimated those that weren’t already available. We then ran computational simulations of this healthy case to determine the abundance of our model’s metabolites, and compared the numerical results to the available experimental data. The close match between our numerical results and the experimental measurements validated our model (see Fig. 5).

The main motivation for building this model was to be able to analyse the effects in the concentration of all the considered metabolites when [GLUT1] was modulated for a greater number of values than in the previously analyzed experimental cases (GLUT1 at 100%, 50%, and less than 30% of the healthy concentration). Glucose deficiency has been suggested to cause cone photoreceptor cell death in patients with rod dystrophies. Our objective was to gain insight on how the cells’ regulating mechanisms maintain homeostasis in healthy and compromised conditions. To this end, we defined the parameter [GLUT1] which represents the proportion of GLUT1 availability, relative to the healthy case (represented by [GLUT1] = 1). We ran simulations with [GLUT1] = (1, 0.44, and 0.18) and compared the numerical steady state results to the available experimental data. The response of our model to a variation of [GLUT1] was validated by the close match between the numerical results and the experimental measurements (see Fig. 6).

Then, we simulated our model with [GLUT1] = 1 and gradually decreased the parameter’s value to zero in 100 equal steps (0.01 decrease in each step), allowing the system to reach a steady state. By the analysis of all the different steady state concentrations obtained, we found that the regulating mechanisms in the rods act to preserve some of the cell’s functions, while some others are neglected. As [GLUT1] decreases the metabolites concentrations that are largest are considered to be most favored. In particular, we noticed that the production of G3P in the KP , and the the production of LACT, G6P and F16BP, which are created during aerobic glycolysis (see right panel of Fig. 7) are favored. This suggested that the rods prioritize their own survival by ensuring their OS renewal (mostly enabled by G3P) and they also try to maintain aerobic glycolysis sacrificing the NADPH production in the PPP. Interestingly, pyruvate (the fuel for the rods’ OXPHO) also decays very quickly. However, when [GLUT1] is near complete exhaustion and not enough glucose can enter the rods to maintain aerobic glycolysis and the G3P production, all the metabolite concentrations collapse to zero, which represents a critical transition to the ceasing of all the cells’ functions and its consequential death. Meanwhile, as the value of [GLUT1] decreased, we observed a great increase in one of the intermediate products of the RPE’s glycolysis, F16BP, and a permanence close to its nominal value in the other, G6P (see left panel of Fig. 7). This, along with the slow but steady decline of the RPE’s pyruvate concentration for values of [GLUT1] less than 0.5, corresponding to the rods’ lactate level of approximately 0, led us to conclude that this cell, which in healthy conditions is highly dependent on the rods, is capable of surviving independently of rods’ lactate contribution by changing the way it functions, as long as it can uptake enough glucose. When the RPE’s glucose intake is insufficient to maintain this alternative mode of functioning, which happens when [GLUT1] is extremely low, all the RPE’s metabolite concentrations collapse to zero. This critical transition represents the death of the cell.

The mathematical model presented here offers many possibilities to explore the effects of changes in any of its parameters, each of which represents a physiological characteristic of the RPE-rod unit, enabling the analysis of its behavior under many different conditions beyond the ones explored here. For example, the mechanisms’ response to perturbations, and their relative contribution to promote survival can be tested. An interesting perturbation that can easily be implemented by changing only one parameter is a hyper or hypoglycemic state. In Harder et al.^61^ the impact of disturbing glucose and pyruvate levels on metabolism when intra-ocular pressure is elevated is demonstrated. Moreover, oral pyruvatye administration is proposed as a treatment that protects from neurodegeneration in models of glaucoma. Yet, the exact mechanism by which the treatment contributes to protect sight is not completely known. Another direction of future work could be to introduce a variable representing oxygen availability, and exploring the behaviour of the system when its gradient is changed, comparing it to existing data from experiments in mice^62,63^. The model presented here offers the opportunity to shed light to these mechanisms at the cost of finding suitable sets of parameters. This cost is low when compared to the resources that wet experiments covering a wide range of conditions would require. We envisage that the numerical data produced by conducting in silico experiments using this model will help experimentalists prioritize and narrow down new experiments, leading to a more effective use of lab resources. Experimental discoveries, in turn, would help tune and add to the functionalities of the model. This work is a first approximation to building a mathematical and computational model of the lactate-mediated metabolic exchange in the retina. Future possible work includes the addition of pathways that are not included here such as OXPHO, TCA cycle, photoreceptors’ lactate uptake, etc, or other types of cells (for example, the Muller cells). We expect that this model will grow in components and functionality along with new experimental findings.

## Methods

All the simulations, plots and statistical analysis wereperformed using Python. For the numerical integration we used the Real-valued Variable-coefficient Ordinary Differential Equation solver odeint from scipy.integrate. For all the plots we used matplotlib.

## Supplementary Information

Supplementary Information.

## Abbreviations

RPE: Retinal pigmented epithelium
HIV: Human immunodeficiency virus
TCA: Tricarboxylic acid cycle
DHAP: Dihydroxyacetone phosphate
GDS: GLUT1 deficiency syndrome
SLC2A1: Solute carrier family 2 member 1
MCT1: Monocarboxylate transporter 1
PPP: Penthose phosphate pathway
LDHB: Lactate dehydrogenase B
ROS: Reactive oxidative species
OXPHOS: Oxidative phosphorylation
PEP: Phosphoenol pyruvate
GLUT1: Glucose transporter 1
ATP: Adenosine triphosphate
HK2: Hexokinase-2
RdCVF: Rod-derived cone viability factor
SLC1A1: Solute carrier family 1 member 1
OS: Outer segments
MCT3: Monocarboxylate transporter 3
KP: Kennedy pathway
NADPH: Nicotinamide adenine dinucleotide phosphate
G3P: Glyceraldehyde 3-phosphate
HKII: Hexokinase 2
