# Supplementary material for: A mathematical model of GLUT1 modulation in rods and RPE and its differential impact in cell metabolism
Source: Sci Rep. 2022 Jun 23;12:10645. doi: 10.1038/s41598-022-13950-3 (PMC9226191; doi:10.1038/s41598-022-13950-3)
Supplement: Supplementary file 1 — Supplementary Information. [file 41598_2022_13950_MOESM1_ESM.pdf]

—Supplementary Information—

A mathematical model of GLUT1 modulation in rods and RPE and its differential impact in cell metabolism

Andrea Aparicio *[1], Erika T. Camacho[1], Nancy J. Philp[2], and Stephen A. Wirkus[1]

[1]*School of Mathematical and Natural Sciences, Arizona State University, Glendale, AZ*
[2]*Department of Pathology, Anatomy and Cell Biology, Thomas Jefferson University, Philadelphia, PA*

1 Unit conversion of experimental data

Table S1 contains reported data[1] for the concentrations of lactate in the retina and pyruvate in the retina and the RPE. Note that these values are in units of pMoles per microgram of protein.

**Time** (minutes)	**Retinal lactate** (pM$\cdot\mu$g^{-1})	**Retinal pyruvate** (pM$\cdot\mu$g^{-1})	**RPE pyruvate** (pM$\cdot\mu$g^{-1})
0	0	0	0
5	49.78	1.10	1.33
30	68.28	1.08	0.94
60	99.38	1.23	1.48
120	98.34	1.80	1.12

Table S1: Experimental concentrations of lactate in the retina and pyruvate in the retina and the RPE.[1]

A protein concentration of 20 mg/ml has been previously reported.[2] We used this quantity as a conversion factor in order to express the available experimental data in terms of our model's units, mM/ml. Below we show an example of the conversion, for the lactate concentration at 5 min:

$$49.78\,\frac{pM}{\mu g}\left(\frac{1e^{-9}mM}{1pM}\right)\left(\frac{\mu g}{1e^{-3}mg}\right)\left(20\frac{mg}{ml}\right) = 99.56\text{E-}5\frac{mM}{ml}.$$

Table S2 contains the data of Table S1 converted to mM/ml units. Figure 5depicts a plot of this data.

*correspondence: andrea.aparicio@asu.edu

**Time** (minutes)	**Retinal lactate** (mM·ml^{-1})	**Retinal pyruvate** (mM·ml^{-1})	**RPE pyruvate** (mM·ml^{-1})
0	0	0	0
5	99.56 E-5	2.20 E-5	2.66E-5
30	136.56 E-5	2.16 E-5	1.88 E-5
60	198.76 E-5	2.46 E-5	2.96 E-5
120	196.68 E-5	3.60 E-5	2.24 E-5

Table S2: Experimental concentrations of lactate in the retina and pyruvate in the retina and the RPE in mM·ml^{-1}.[1]

2 Normalization of data

In order to adequately compare our numerical data to the available experimental data, we have re-scaled the the data points to get their relative magnitudes with respect to a particular data point. In the following we provide step by step examples of this procedure.

Let the available data set be conformed by measurements at different times t of a substance present in several mice m, as shown in Table S3. For simplicity, let's assume that these quantities are dimensionless.

	t_1	t_2	t_3
m_1	10	15	20
m_2	8	9	10
m_3	10	10	1

Table S3: Example data

For the first example we will normalize the data with respect to m_1 at time t_1, that is, get the relative measurements with respect to mouse 1 at time 1. To this end, we divide all the data points by the value of m_1 at t_1, i.e., by 10, and then multiply them all by 100. This yields the relative data in Table S4.

	t_1	t_2	t_3
m_1	100	150	200
m_2	80	90	100
m_3	100	100	10

Table S4: Relative data with respect to m_1 at t_1

The next example normalizes the data to obtain each individual mouse's relative measurements with respect to their value at time t_3. Starting with m_1, we divide all three data points by the value at t_3, i.e., by 20, and then multiply them all by 100. An identical procedure should be carried out for the two other mice, yielding the relative data of Table S5

	t_1	t_2	t_3
m_1	50	75	100
m_2	80	90	100
m_3	1000	1000	100

Table S5: Relative data with respect to t_1

Finally, we normalize the data to obtain relative measurements at every time with respect to mouse 1. In a similar way as before, we use the value of m_1 at t_1 to calculate the relative values of m_2 at t_1 and m_3 at t_1 with respect to it, and repeat the procedure for t_2 and t_3. This yields the data relative to m_1 in Table S6

	t_1	t_2	t_3
m_1	100	100	100
m_2	80	60	50
m_3	100	66.66	0.05

Table S6: Relative data with respect to m_1

3 Independence of initial conditions

Often, the solutions of differential equations such as those that comprise the model are dependent on their initial conditions. In the context of our mathematical model this would mean that the steady state concentration of the metabolites could depend on their initial value and that even slightly different concentrations at the beginning of an experiment could lead to a very different final steady state. The goal of our model is to trace the impacts of varying a specific parameter in the steady state concentration of the cells' metabolites and thus, we need to make sure that this steady state is not dependent of the initial conditions and is only sensitive to the parameter variation. To this end, we fixed all the parameters in the model to the values in Table 1, except for the initial conditions, and ran simulations with a wide range of them. The maximal numerical value for any of the metabolite concentrations in Figure 7 is of approximately 400$\%$ of the nominal case and thus we defined the range of initial conditions as 1$\%$ to 500$\%$ of the nominal ones (that appear in Table 1). Each simulation was run for 1500 time steps, to ensure that a steady state was reached even for large or very small initial conditions, i.e., far from their nominal value. We observed that the solutions reached the exact same steady state for each one of the initial conditions in the mentioned range, for all the measured metabolites. This shows that the steady state of the system is unique and independent of the initial conditions for the tested range and parameters, which represents the spectrum of the physiological conditions that we consider in our experiments.

4 Parameter sensitivity

To illustrate the sensitivity of our model in terms of approximation to the equilibrium and steady state value, to variations of the kinetic parameters used in Table 1, we systematically ran the simulations changing every parameter by a small quantity. Figures SI1, SI2, and SI3 show the results of these simulations and were built as follows: The black lines represent the resulting concentration of the metabolites when the parameters are as they appear in Table 1. The colored lines represent the

resulting concentration of the metabolites when all the parameters are fixed at their value in Table 1, except for the parameter indicated on top of the panel which is varied between 20% (cyan) and 200% (magenta) of its value in Table 1. We notice that the model is not very sensitive to changes in the K_m values, with a few exceptions, while many metabolites are very sensitive to changes in the V_{max} values. In particular, the steady state concentrations of all the metabolites are sensitive to variations of the $V_{max_{[\mathrm{gr}]}}$ value. This is expected because the glucose that is used in both cells is uptaken by the rods and then directed to the RPE. Therefore, this value must be chosen carefully.

Figure SI1

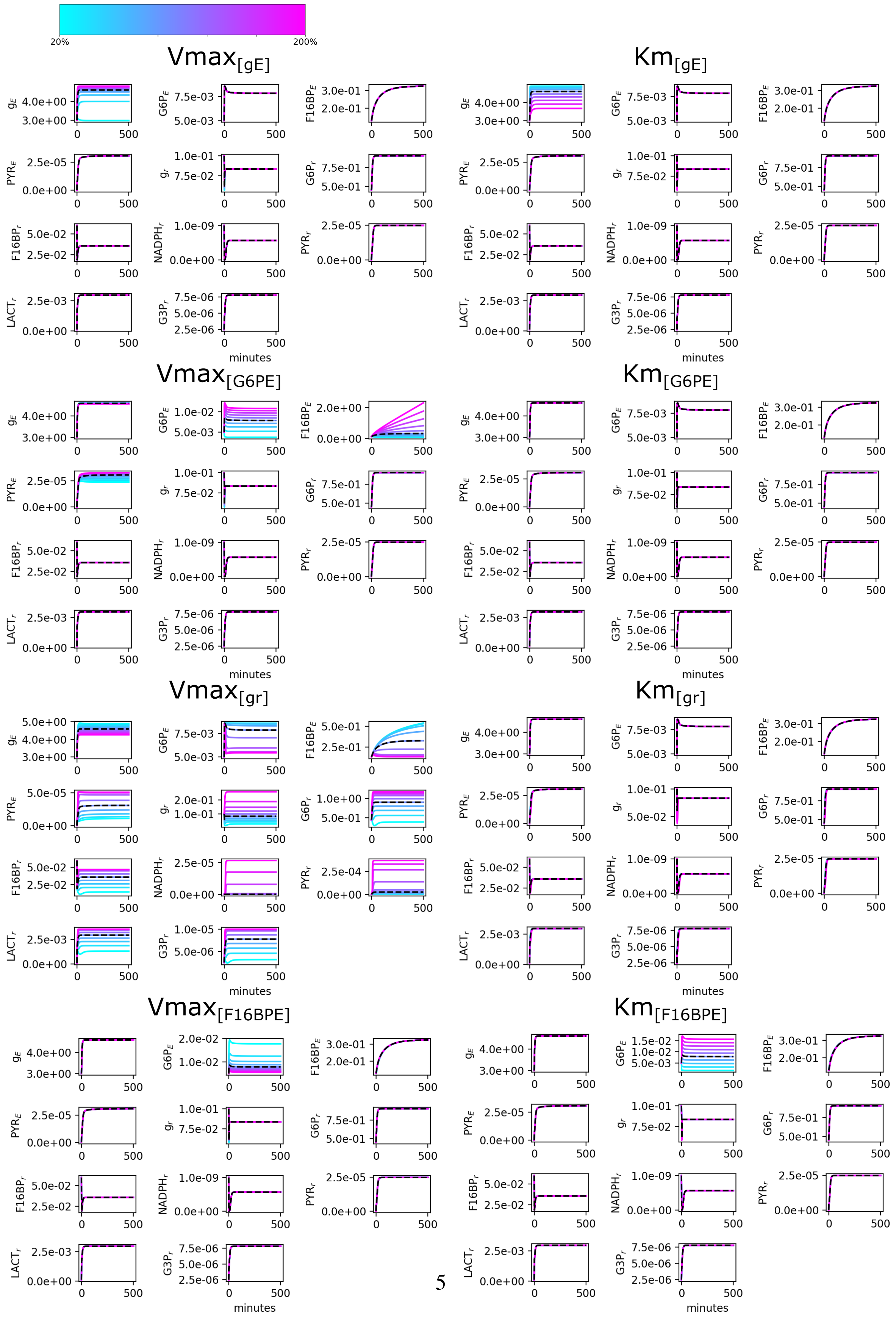
20%
200%
Vmax[gE]
Km[gE]
Vmax[G6PE]
Km[G6PE]
Vmax[gr]
Km[gr]
Vmax[F16BPE]
Km[F16BPE]
minutes

Figure SI2

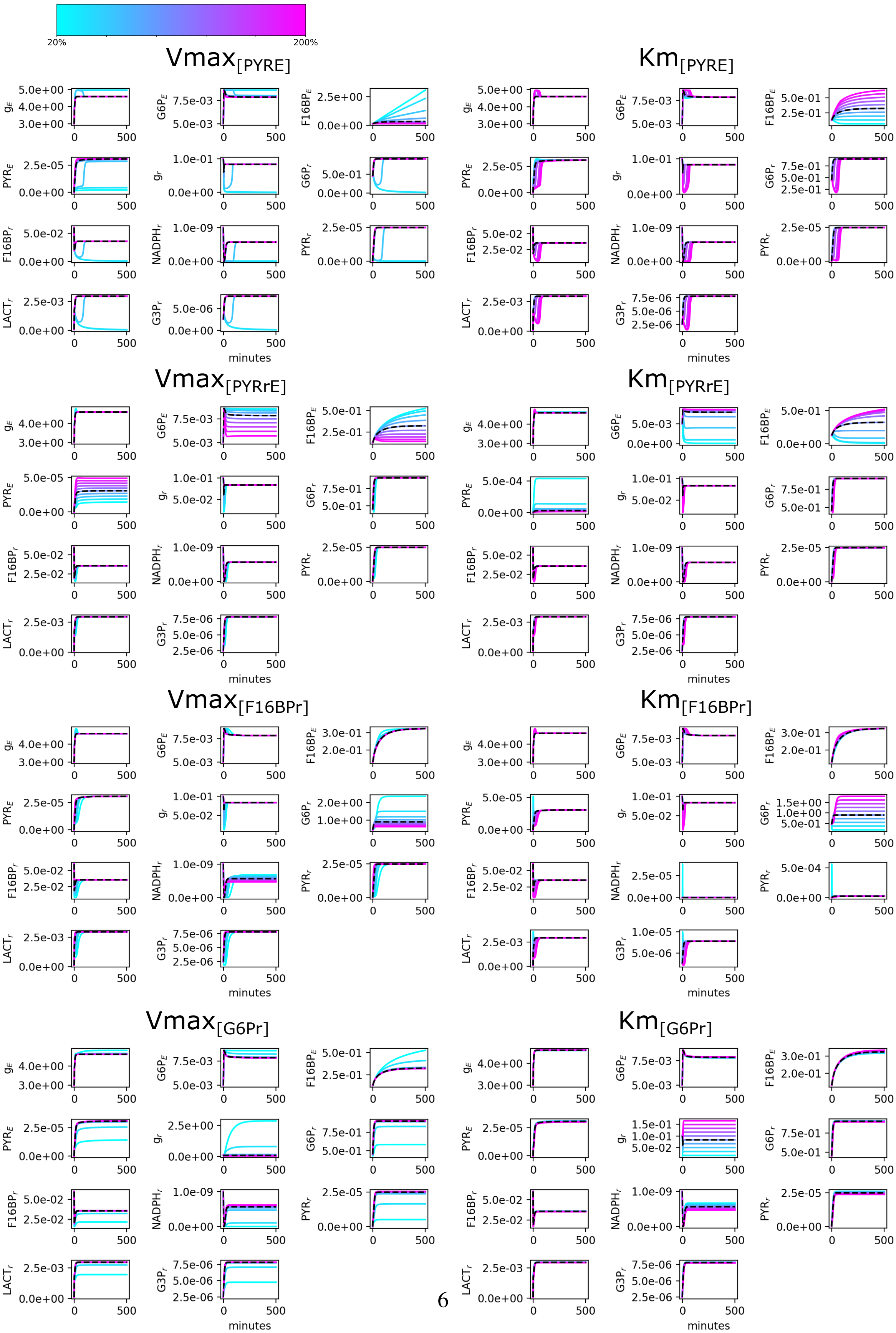

20%
200%
Vmax[PYRE]
Km[PYRE]
Vmax[PYRrE]
Km[PYRrE]
Vmax[F16BPr]
Km[F16BPr]
Vmax[G6Pr]
Km[G6Pr]
minutes

Figure SI3

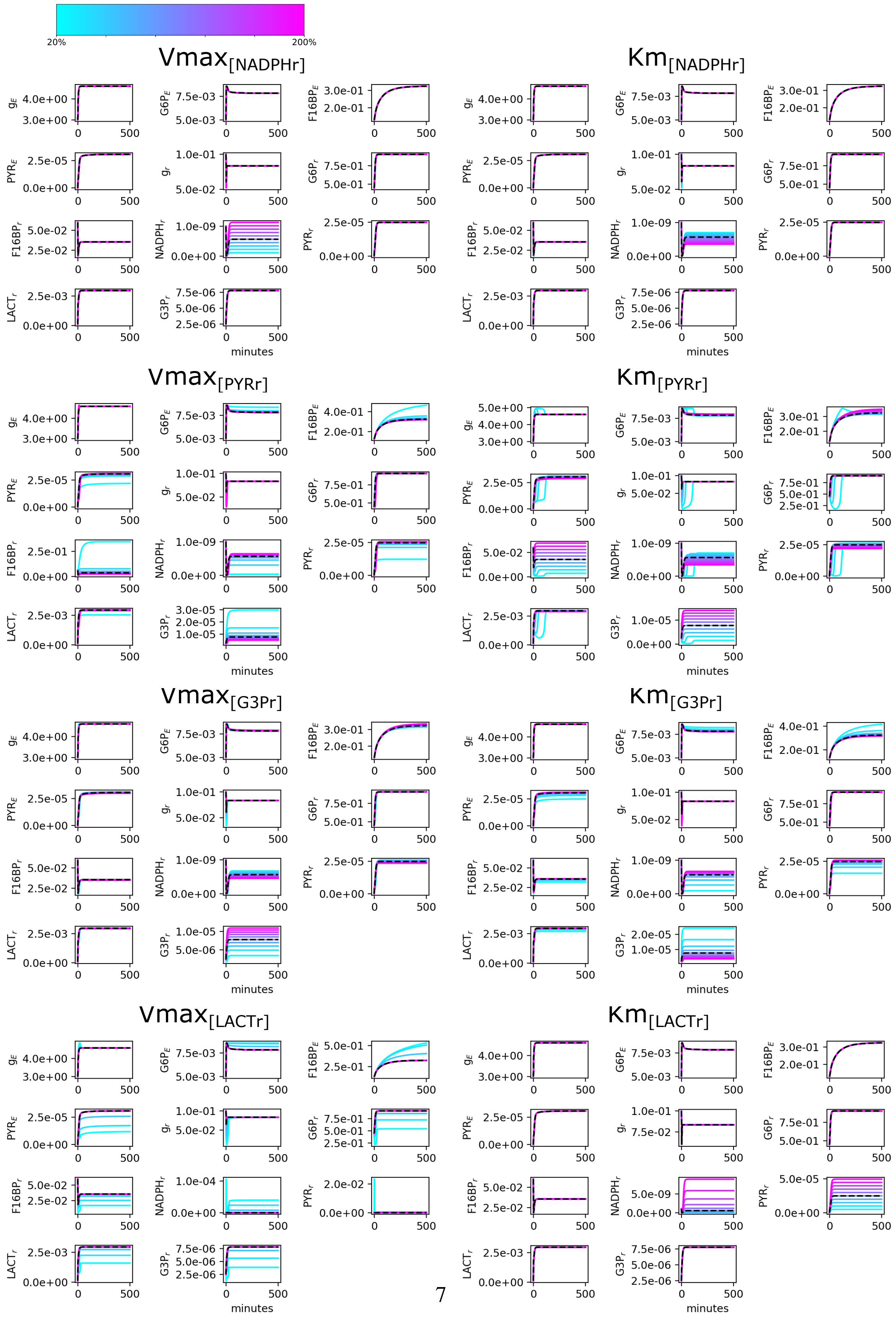

20%
200%
Vmax[NADPHr]
Km[NADPHr]
Vmax[PYRr]
Km[PYRr]
Vmax[G3Pr]
Km[G3Pr]
Vmax[LACTr]
Km[LACTr]
g_E
$G6P_E$
$F16BP_E$
PYR_E
g_r
$G6P_r$
$F16BP_r$
$NADPH_r$
PYR_r
$LACT_r$
$G3P_r$
minutes

References

[1] Mark A Kanow, Michelle M Giarmarco, Connor SR Jankowski, Kristine Tsantilas, Abbi L Engel, Jianhai Du, Jonathan D Linton, Christopher C Farnsworth, Stephanie R Sloat, Austin Rountree, et al. Biochemical adaptations of the retina and retinal pigment epithelium support a metabolic ecosystem in the vertebrate eye. Elife, 6:e28899, 2017.

[2] Jianhai Du, Whitney Cleghorn, Laura Contreras, Jonathan D Linton, Guy C-K Chan, Andrei O Chertov, Takeyori Saheki, Viren Govindaraju, Martin Sadilek, Jorgina Satrústegui, et al. Cytosolic reducing power preserves glutamate in retina. Proceedings of the National Academy of Sciences, 110(46):18501–18506, 2013.
